# CENPL, ISG20L2, LSM4, MRPL3 are four novel hub genes and may serve as diagnostic and prognostic markers in breast cancer

**DOI:** 10.1038/s41598-021-95068-6

**Published:** 2021-08-02

**Authors:** Jinbao Yin, Chen Lin, Meng Jiang, Xinbin Tang, Danlin Xie, Jingwen Chen, Rongqin Ke

**Affiliations:** 1 School of Medicine, Huaqiao University, Quanzhou, 362021 Fujian China; 2 Department of Pathology, Guangdong Medical University, Dongguan, 523808 Guangdong China

**Keywords:** Breast cancer, Cancer

## Abstract

As a highly prevalent disease among women worldwide, breast cancer remains in urgent need of further elucidation its molecular mechanisms to improve the patient outcomes. Identifying hub genes involved in the pathogenesis and progression of breast cancer can potentially help to unveil mechanism and also provide novel diagnostic and prognostic markers. In this study, we integrated multiple bioinformatic methods and RNA in situ detection technology to identify and validate hub genes. EZH2 was recognized as a key gene by PPI network analysis. CENPL, ISG20L2, LSM4, MRPL3 were identified as four novel hub genes through the WGCNA analysis and literate search. Among these, many studies on EZH2 in breast cancer have been reported, but no studies are related to the roles of CENPL, ISG20L2, MRPL3 and LSM4 in breast cancer. These four novel hub genes were up-regulated in tumor tissues and associated with cancer progression. The receiver operating characteristic analysis and Kaplan–Meier survival analysis indicated that these four hub genes are promising candidate genes that can serve as diagnostic and prognostic biomarkers for breast cancer. Moreover, these four newly identified hub genes as aberrant molecules in the maintenance of breast cancer development, their exact functional mechanisms deserve further in-depth study.

## Introduction

Breast cancer is one of the most common malignant tumors that present serious and major threats to female life and health. Although current breast cancer therapeutic methods have been well developed and improved, latest data showed that breast cancer still has a high mortality rate among women worldwide. Thus, there is still an urgent need to explore the potential molecular mechanisms for improving the patient outcomes^1,2^.

In the past decade, applications of high-throughput chip and sequencing technologies have resulted in accumulation of a wealth of novel research data resource that can be analyzed by a series of bioinformatic methods, providing a novel approach to explore the molecular mechanism of tumorigenesis and tumor development^3^. Among a wide range of different bioinformatics tools, weighted gene co-expression network analysis (WGCNA) algorithm is the most commonly used method for gene co-expression network research. By constructing co-expression gene modules and associating external information, the key gene modules and potential hub genes can be identified^4–6^. In general, hub genes show high connectivity in the gene co-expression network, which often located in the upstream of the gene regulatory network and play a predominant role in the gene network coordination^7,8^. Therefore, identification of potential novel hub genes is of great significance for exploring the mechanism of tumor initiation and progression. Selection of appropriate dataset is an important prerequisite for screening hub genes, and multiple types of datasets that were generated from different platforms are now available in the public database^9^. To explore the best potential of these datasets, it would be of great advantage to integrate them for downstream analysis. To achieve this goal, we used Robust Rank Aggregation (RRA) analysis algorithm for the process of breast cancer datasets. RRA is a reliable bioinformatic method that can remove substantial inter-study variations and statistical analysis difficulties existed in individual studies via integrating the gene expression profiles of different cross-platform datasets^10^. It has been used in various malignant tumor studies, such as in hepatocellular carcinoma, colorectal cancer, lung cancer and thyroid cancer^11–14^. Heterogeneity is one of the characteristics of tumor cells, which is reflected by different expression patterns of genes at the transcription level^15^. Analyzing and identifying the temporal and spatial heterogeneity information of RNA expression can be of great value to reveal the structural relationship between tissues and cells, as well as to uncover the potential functions of genes in disease state. RNA in situ detection technology can be used for studying the heterogeneity of RNA expression, and under the condition of maintaining tissue and cell morphology integrity, it can obtain the spatial localization and abundance of intracellular RNA at the single cell level^16^.

In this study, we first integrated DEGs from multiple breast cancer datasets based on RRA algorithms, and then identify the key gene of known functions using PPI network analysis. At the same time, WGCNA algorithm was applied to construct a weighted gene co-expression network and screen for potential novel hub genes related to breast cancer. The diagnostic performance and prognostic value of these novel hub genes were evaluated and their possible molecular mechanisms in breast cancer were explored by bioinformatic methods. Finally, we also made full use of RNA in situ detection technology to detect the expression abundance and spatial localization of each hub gene at single cell level, and further analyzing the expression differences and correlations, such that to validate those results from the bioinformatic analysis mentioned above.

## Results

### Identification of robust DEGs in breast cancer by the RRA analysis

Differentially expressed genes (DEGs) of eight datasets from the GEO database were integrated to perform RRA analysis, and the characteristics for each dataset are shown in Table 1. We used |log2FC|> 1 and *p* value < 0.05 as screening criteria to obtain the robust DEGs between breast cancer tissues and normal tissues. A total of 512 robust DEGs were identified, containing 202 up-regulated genes and 310 down-regulated genes (Supplementary Table S1). Supplementary Fig. S1 shows the top 20 most significant up-regulated and down-regulated robust DEGs obtained by RRA methods from these eight different datasets. Of those, COL11A1 (*P* = 2.47E−19, adjusted *P* = 6.53E−15, logFC = 2.86) and S100P (*P* = 1.24E−17, adjusted *P* = 3.28E−13, logFC = 3.50) were the two most significant up-regulated genes. Meanwhile, LEP (*P* = 2.68E−14, adjusted *P* = 7.06E−10, logFC = − 3.12) and FGF2 (*P* = 2.84E-14, adjusted *P* = 7.48E−10, logFC = − 1.87) were the two most significant down-regulated gene in breast cancer tissues.

**Table 1:** Characteristics of the included GEO datasets.

Dataset ID	Country	Normal	Tumor	platform ID	Number of rows per platform
GSE21422	Germany	5	14	GPL570	54,675
GSE33447	China	4	12	GPL14550	42,545
GSE42568	Ireland	17	104	GPL570	54,675
GSE14999	Italy	61	68	GPL3991	23,653
GSE65194	France	11	153	GPL570	54,675
GSE15852	Malaysia	43	43	GPL96	22,283
GSE5764	Czech Republic	20	10	GPL570	54,675
GSE3744	USA	7	40	GPL570	54,675
In total		168	444		

### GO functional enrichment analysis and KEGG pathway enrichment analysis of robust DEGs

**Figure 1:**
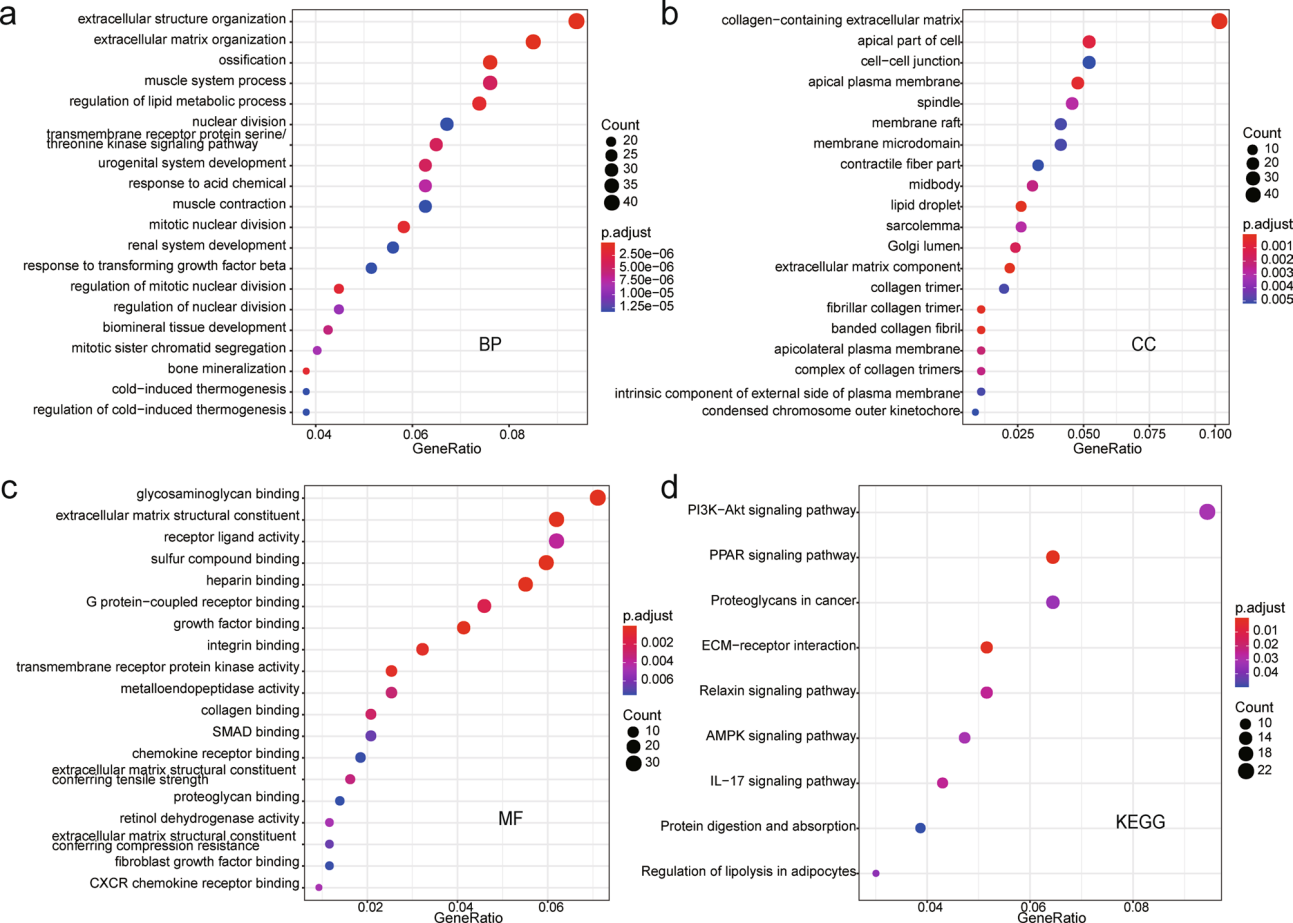
GO enrichment analysis and KEGG pathways analysis of 512 DEGs. (**a)** GO terms of biological process (BP); (**b)** GO terms of cellular component (CC); (**c)** GO terms of molecular function (MF); (**d)** KEGG pathways terms.

To gain insight into the known biological processes and pathways involved in breast cancer, GO functional enrichment analysis and KEGG pathways analysis of 512 robust DEGs were performed. The results showed that those robust DEGs were significantly enriched in 720 GO terms and 9 KEGG pathways, respectively (Supplementary Table S2). Figure 1a–c show the top 20 GO terms, GO terms related to biological process including extracellular structure organization, extracellular matrix organization, ossification, mitotic nuclear division, and regulation of lipid metabolic process (Fig. 1a); Cellular component GO terms were mainly distributed in collagen − containing extracellular matrix, extracellular matrix component, lipid drople and fibrillar collagen trimer (Fig. 1b). The molecular function GO terms consisting of extracellular matrix structural constituent, glycosaminoglycan binding, heparin binding, sulfur compound binding growth factor binding those DGEs were significantly enriched (Fig. 1c). What′s more, KEGG pathway enrichment analysis revealed that PI3K-AKT signaling pathway, PPAR signaling pathway, ECM − receptor interaction, Relaxin signaling pathway, IL − 17 signaling pathway, AMPK signaling pathway were significantly associated with these robust DEGs identified. (Fig. 1d).

### EZH2 as a key gene by PPI network analysis

The PPI network of the 512 DEGs, including 493 nodes and 2993 edges was constructed via STRING database (minimum required interaction score: 0.4). By ranking the PPI network nodes using 9 topological analysis methods including both local- and global-based algorithms from cytoHubba plugin of Cytoscape software, we found the EZH2 score ranked in the top 10 by 9 algorithms (Table 2). Furthermore, by performing gene module analysis using MCODE plugin in Cytoscape software, EZH2 gene was also found in Module 1 (Supplementary Fig. S2), which is the most important module (MCODE score = 31.942) among all modules. In addition, GEPIA database analysis showed that the mRNA expression levels of EZH2 were significantly higher in breast cancer tissues than normal breast tissues (Supplementary Fig. S3) and its expression level was significantly associated with the poorer prognosis of patients in breast cancer (Supplementary Fig. S4). GSEA demonstrated that EZH2 high expression level group was significantly enriched in “Cell cycle” and “DNA replication”, which are known to be tumor cell proliferation related pathways (Supplementary Fig. S5).

**Table 2:** Hub genes for highly expressed genes ranked by different CytoHubba methods.

category	Rank methods in CytoHubba								
	MCC	MNC	Degree	BottleNeck	EcCentricity	Closeness	Radiality	Betweenness	Stress
1	**EZH2**	FN1	FN1	FN1	FN1	FN1	FN1	FN1	FN1
2	CDK1	CDH1	CDH1	**EZH2**	**EZH2**	CDH1	CDH1	CDH1	CDH1
3	CCNB1	FGF2	FGF2	CDH1	CDH1	FGF2	FGF2	PPARG	PPARG
4	FOXM1	**EZH2**	**EZH2**	ERBB2	ERBB2	MMP9	MMP9	FGF2	MMP9
5	UBE2C	MMP9	MMP9	PPARG	PPARG	PPARG	ERBB2	ERBB2	FGF2
6	AURKA	CDK1	CDK1	FGF2	FGF2	ERBB2	PPARG	MMP9	ERBB2
7	CDKN3	CCNB1	PPARG	IGF1	IGF1	IGF1	IGF1	**EZH2**	**EZH2**
8	RRM2	FOXM1	CCNB1	POSTN	FOS	**EZH2**	SPP1	FOS	FOS
9	ASPM	PPARG	FOXM1	FOS	DMD	SPP1	FOS	IGF1	IGF1
10	TOP2A	AURKA	ERBB2	DMD	MMP9	FOS	**EZH2**	DMD	SPP1

### WGCNA

**Figure 2:**
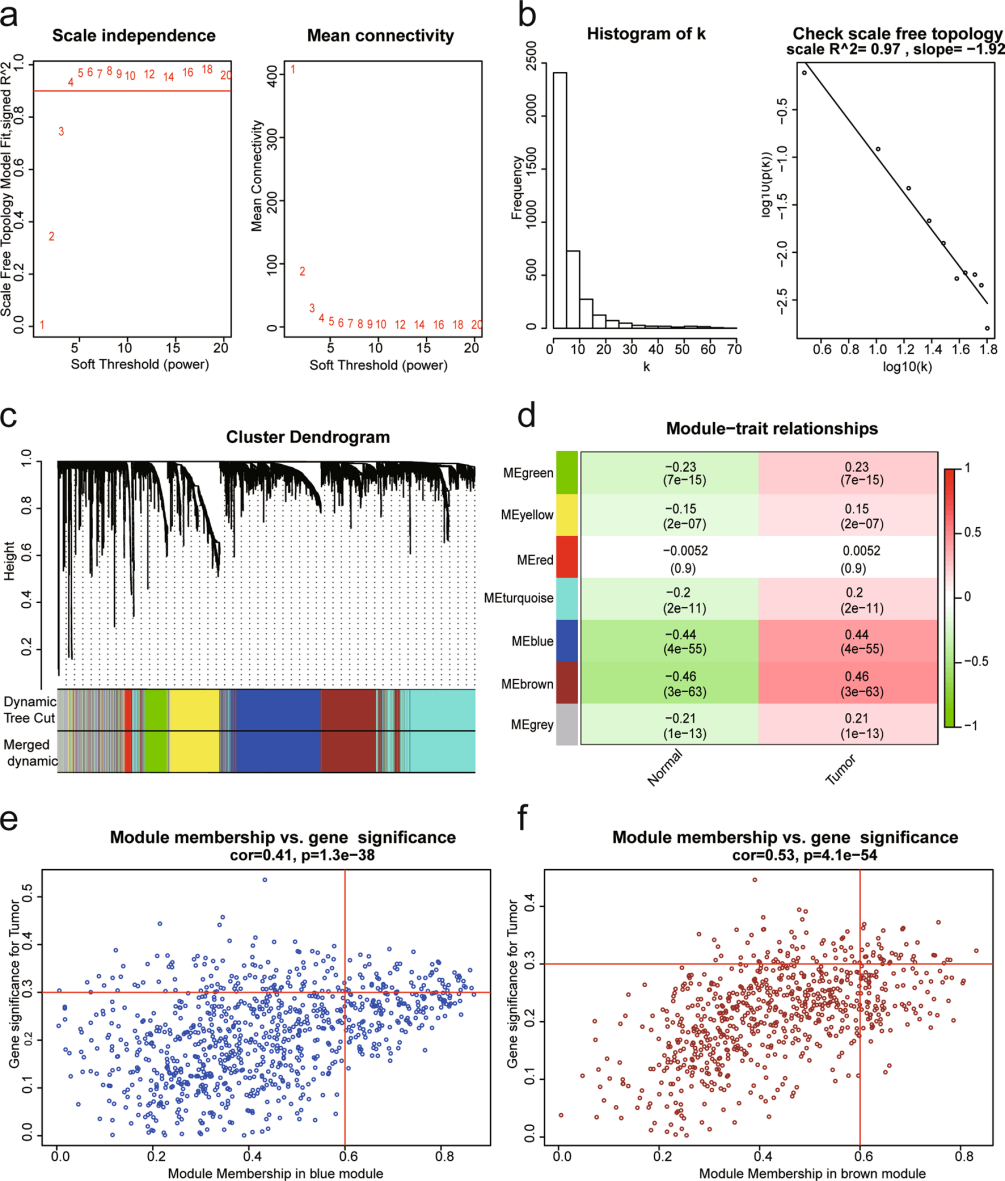
Identification of candidate gene modules and 102 hub genes for breast cancer based on TCGA_BRCA dataset through WGCNA. (**a)** Left: analysis of the scale-free fitting indices for various soft-thresholding powers (β), red line indicated Scale Free Topology Model Fit, signed R^2^ is 0.90. Right: mean connectivity analysis of various soft-thresholding powers (β value range 1–20); (**b)** Left: histogram shows the frequency distribution of the k (namely connection) when β = 5. Right: checking the scale-free topology when β = 5, the figure shows that log10(k) and log10(p(k)) are negatively correlated (correlation coefficient 0.97), denoting that the gene scale-free network that we constructed is guaranteed; (**c)** Clustering dendrograms of genes based on dissimilarity topological overlap calculation formula (1—TOM) and merged gene set modules. Seven weighted gene co-expression network modules were constructed and shown in different colors; (**d)** Heatmap of the correlation between module eigengenes and breast cancer samples traits (Tumor). The numbers in each square of heatmap indicates the Pearson correlation coefficient (up) and P value (down); (**e)** Scatter plot of gene significance for “Tumor” and module membership in the blue module. The red lines indicate MM value = 0.6 and GS value = 0.3; (**f)** Scatter plot of gene significance for “Tumor” and module membership in the brow module. The red lines indicate MM value = 0.6 and GS value = 0.3.

We performed WGCNA using the TCGA_BRCA dataset that incorporate 3,769 up-regulation DEGs (*P* value < 0.05) derived from the above RRA analysis to find key gene modules in breast cancer. After series of quality assessment for gene expression matrix, we set soft threshold as 5 (scale free R^2^ = 0.97, slope =  − 1.92) to construct and validate the scale-free network (Fig. 2a,b). By setting minimal module size as 50 genes and cut height as 0.25 to merge similar modules, seven modules were obtained eventually (Fig. 2c; non-clustering DEGs shown in gray). From the heatmap of module–trait correlations (Fig. 2d), we identified that the blue module (cor = 0.44, *P* = 4e−55) and brown module (cor = 0.46, *P* = 3e−63) were most correlated to breast cancer (Fig. 2e,f). GO functional enrichment analysis and KEGG pathways analysis of genes in blue module and brown module prove our our judgement (Supplementary Table S3-4 showed the GO functional enrichment analysis and KEGG pathways analysis results of genes in blue module and brown module separately). The blue module contained 920 genes and the brown module contained 730 genes. Next, we set the filter standard of hub gene associated with breast cancer: module membership (MM) value > 0.6 and gene significance (GS) value > 0.3, and found that 64 hub genes from the blue module and 38 hub genes from the brown module meet the eligibility criteria (Table 3). By combining with literature searches, four hub genes (CENPL, ISG20L2, MRPL3, and LSM4) were obtained for further analysis. None of these four selected genes had been reported in breast cancer molecular mechanisms studies.

**Table 3:** Hub genes identified in the blue and brown modules associated with breast tumor via WGCNA analysis.

Module	Hub genes
blue	PPM1G, CDCA4, TACC3, CCNF, TIMELESS, SRSF1, CENPU, **MRPL3**, PSMD14, **ISG20L2**, ESRP1, TMEM206, SPC24, ASF1B, ZWINT, CDKN3, UHRF1, MIS18A, ZWILCH, MTHFD2, CKS2, CKAP2, ELAVL1, PCNA, FEN1, DTL, E2F1, H2AFZ, HDGF, PBK, CDC25C, ECT2, TUBA1C, SPC25, TROAP, HMMR, IQGAP3, ESPL1, UBE2T, EZH2, RACGAP1, OIP5, RAD51, CCNB1, LMNB1, NEK2, NUSAP1, GINS1, ERCC6L, **CENPL**, CENPF, DLGAP5, KIFC1, KIF11, KIF20A, CKAP2L, BUB1B, KIF23, PLK1, HJURP, NCAPH, NCAPG, KIF4A, TPX2
brown	TJP3, POLR3K, ATP6V0B, PAFAH1B3, DTYMK, FBXL19, VARS2, TRAF2, VAMP8, **LSM4**, SNRPB, WDR34, CHMP4B, SLC25A39, HMOX2, KCTD13, CDK5, TPRN, RUVBL2, ZDHHC12, DPM2, BAX, TSEN54, AXIN1, TBC1D10B, KIF22, CACFD1, PRR14, NR2F6, COPE, SNRNP25, NUDT16L1, PGP, PPP4C, PHKG2, NMRAL1, ROGDI, MRPS34

Four selected genes are annotated in bold.

### Correlation analysis of the four novel hub genes with clinicopathological variables in breast cancer

**Figure 3:**
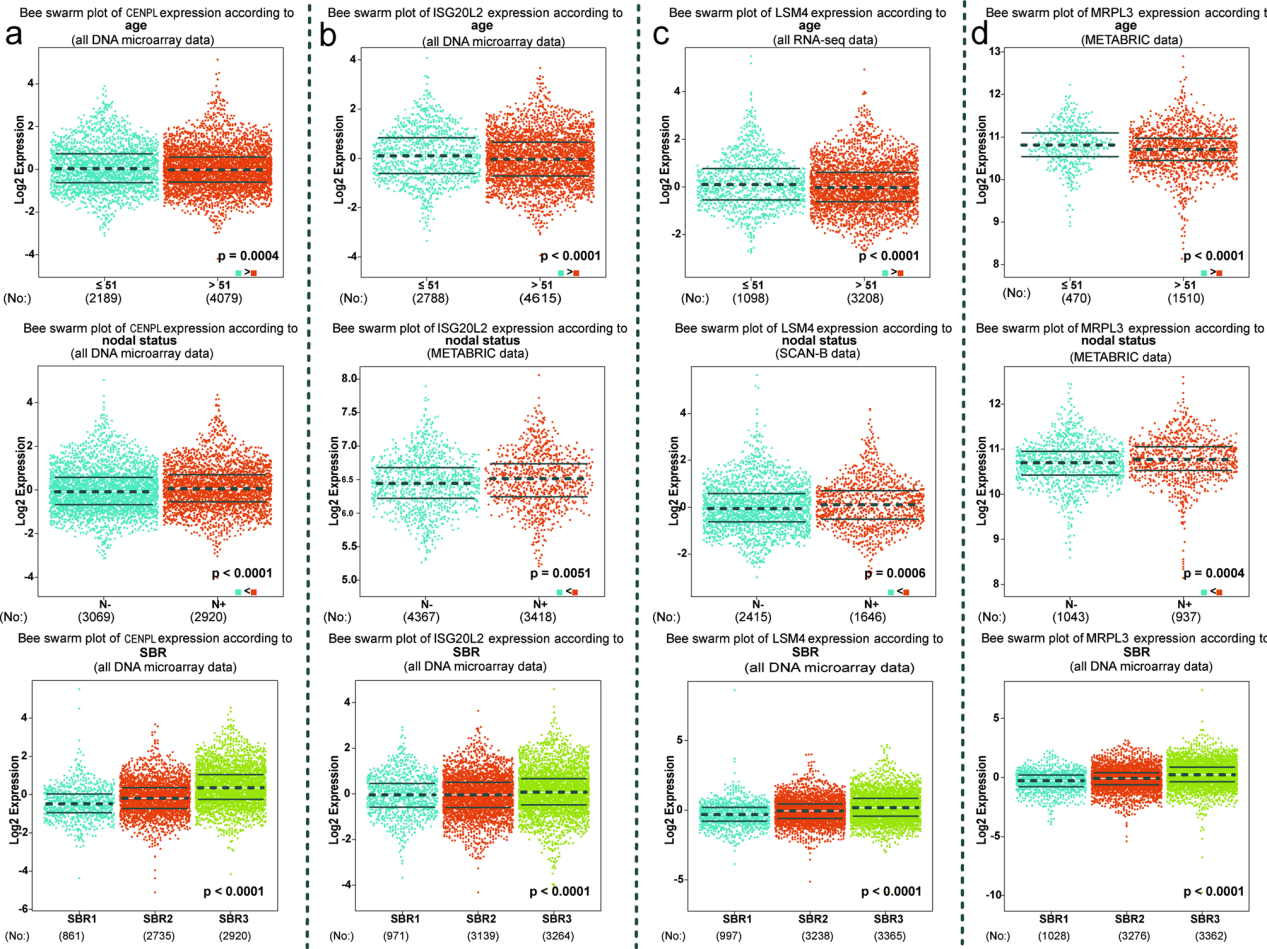
Relationship between four novel key genes expression levels and clinicopathological variables in breast cancer based on bc-GenExMiner platform.

In view of the bc-GenExMiner v.4.6 contains a relatively large number of samples and rich clinical information, we explored the relationships between the expression levels of the novel four hub genes and the clinicopathological variable in this platform. First, we found the expression levels of four novel hub genes (CENPL, ISG20L2, MRPL3, and LSM4) were significantly higher in the subjects aged ≤ 51 years, and high expression of CENPL, ISG20L2, MRPL3, and LSM4 were associated with lymph node metastasis and higher SBR grade (*P* < 0.05, Fig. 3a−c). Moreover, The TNBC and basal-like BC patients both displayed significantly increased expression of CENPL, ISG20L2, MRPL3, and LSM4 than the non-TNBC and non-basal-like patients, and the expression of CENPL, ISG20L2, MRPL3, and LSM4 were also significantly positively related to Ki67 status (Supplementary Fig. S6). Thus, we can conclude that CENPL, ISG20L2, MRPL3, and LSM4 are closely related to clinicopathological variables of BC.

### Validation of the expression differences of the four novel hub genes

**Figure 4:**
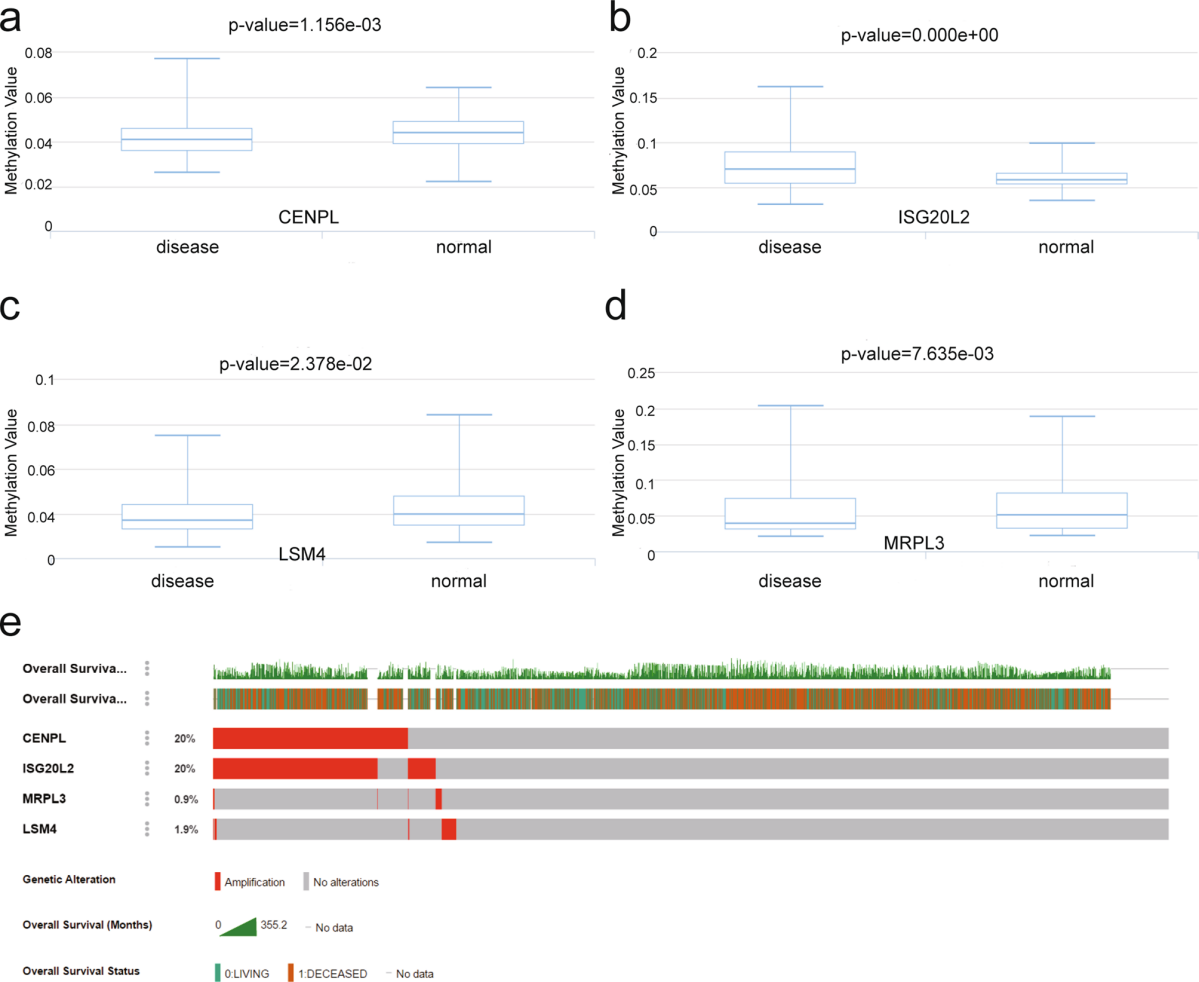
Methylation level analyses and genetic alteration of novel hub genes for breast cancer. (**a–d**) the methylation levels of CENPL, ISG20L2, LSM4, and MRPL3 in breast cancer and normal tissues were examined using DiseaseMeth 2.0 databaset based on 450 k (Illumina Infinium HumanMethylation450 BeadChip) platform; (**e**) Genetic alterations of CENPL, ISG20L2, MRPL3, and LSM4 were examined in cBioPortal database.

Based on the TCGA_BRCA and match TCGA normal and GTEx data of GEPIA database, the mRNA expression levels of these genes (CENPL, ISG20L2, MRPL3 and LSM4) were also significantly higher in breast cancer tissues than normal tissues (Supplementary Fig. S7). Immunohistochemistry analysis from The Human Protein Atlas database also showed that these four hub genes were up-regulated in protein expression level in breast lobular carcinoma (Supplementary Fig. S8). To elucidate the underlying mechanisms of abnormal up-regulation of these four hub genes in breast cancer, we first investigated the association between gene expression and their methylation levels. DiseaseMeth version 2.0 analysis displayed that the mean methylation levels of CENPL, MRPL3 and LSM4 were all significantly reduced in breast cancer compared to normal breast tissues (*P* < 0.05) (Fig. 4a,c,d). While the mean methylation levels of ISG20L2 significantly increased in breast cancer compared to normal breast tissues (*P* < 0.05) (Fig. 4b). Additionally, genetic alterations of CENPL, ISG20L2, MRPL3, and LSM4 were further examined in cBioPortal database, showing these four hub genes were altered in 570 (26%) of 2173 breast cancer patients (Fig. 4e). CENPL and ISG20L2 showed the highest alteration levels (20%) with gene amplification as the main alteration type.

### Identifying the diagnostic performance of each hub gene in breast cancer

**Figure 5:**
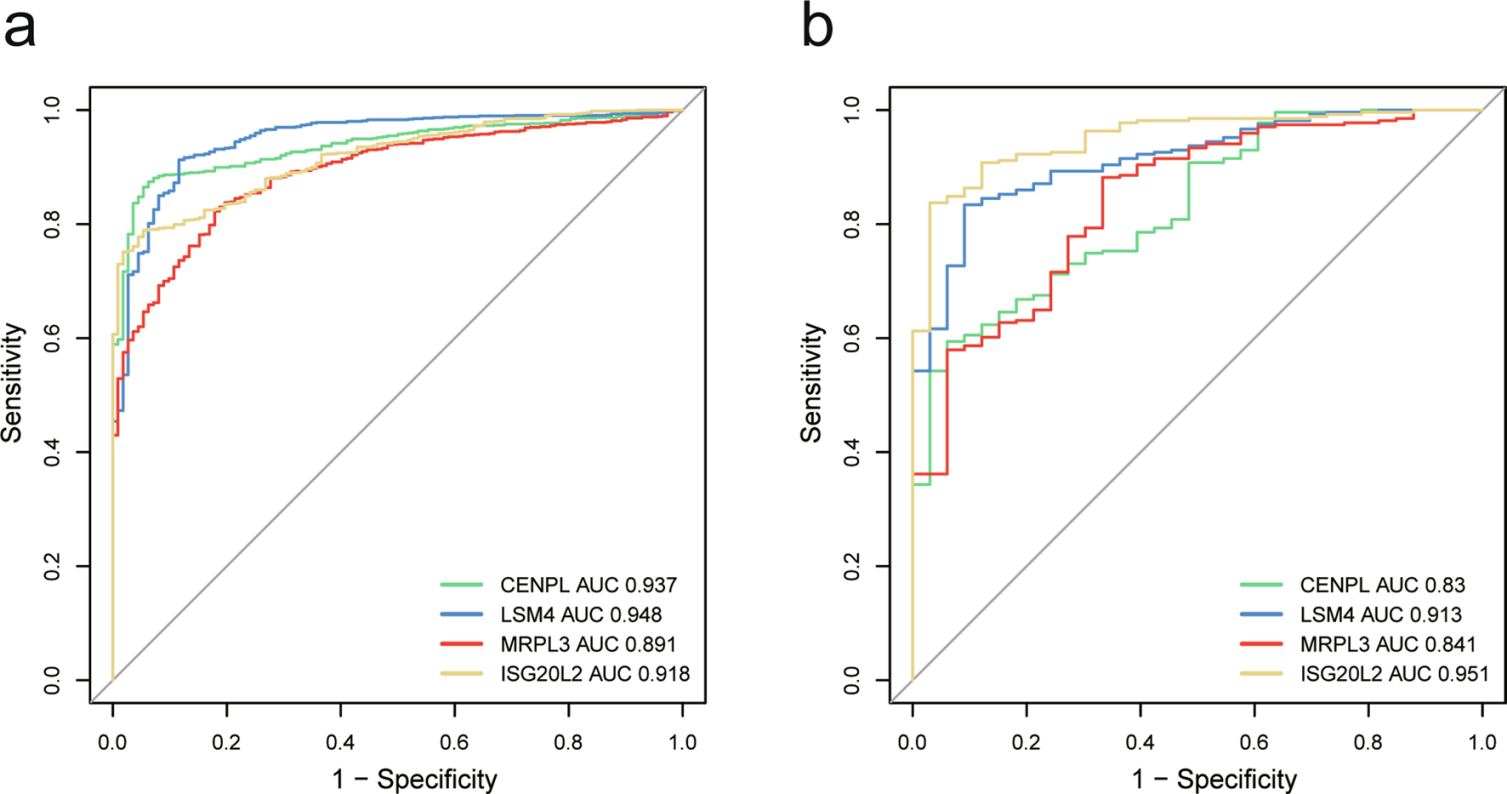
The diagnostic value analysis and validation of four novel hub genes in breast cancer. ROC curves analysis for CENPL, ISG20L2, LSM4 and MRPL3 based on (**a)** TCGA dataset, (**b)** GEO_BRCA dataset. Abbreviation: ROC receiver operating characteristic, AUC area under the ROC curve.

To explore the diagnostic performance of each hub gene in breast cancer, we first performed ROC analysis to assess the diagnostic performances of the four hub genes for detecting breast cancer using TCGA_BRCA dataset, and their AUC values (CENPL AUC: 0.934, LSM4 AUC: 0.948, MRPL3 AUC: 0.891, ISG20L2 AUC: 0.918) were showed in Fig. 5a. These results indicate their good diagnostic performance. Subsequently, ROC analysis in GEO datasets further validate the diagnostic value of these four hub genes. The AUC values of CENPL, LSM4, MRPL3 and ISG20L2 are 0.83, 0.913, 0.841 and 0.951 respectively (Fig. 5b**)**, meaning that the four hub genes all possess good diagnostic performance.

### Prognostic value analysis of each hub gene in breast cancer patients

**Figure 6:**
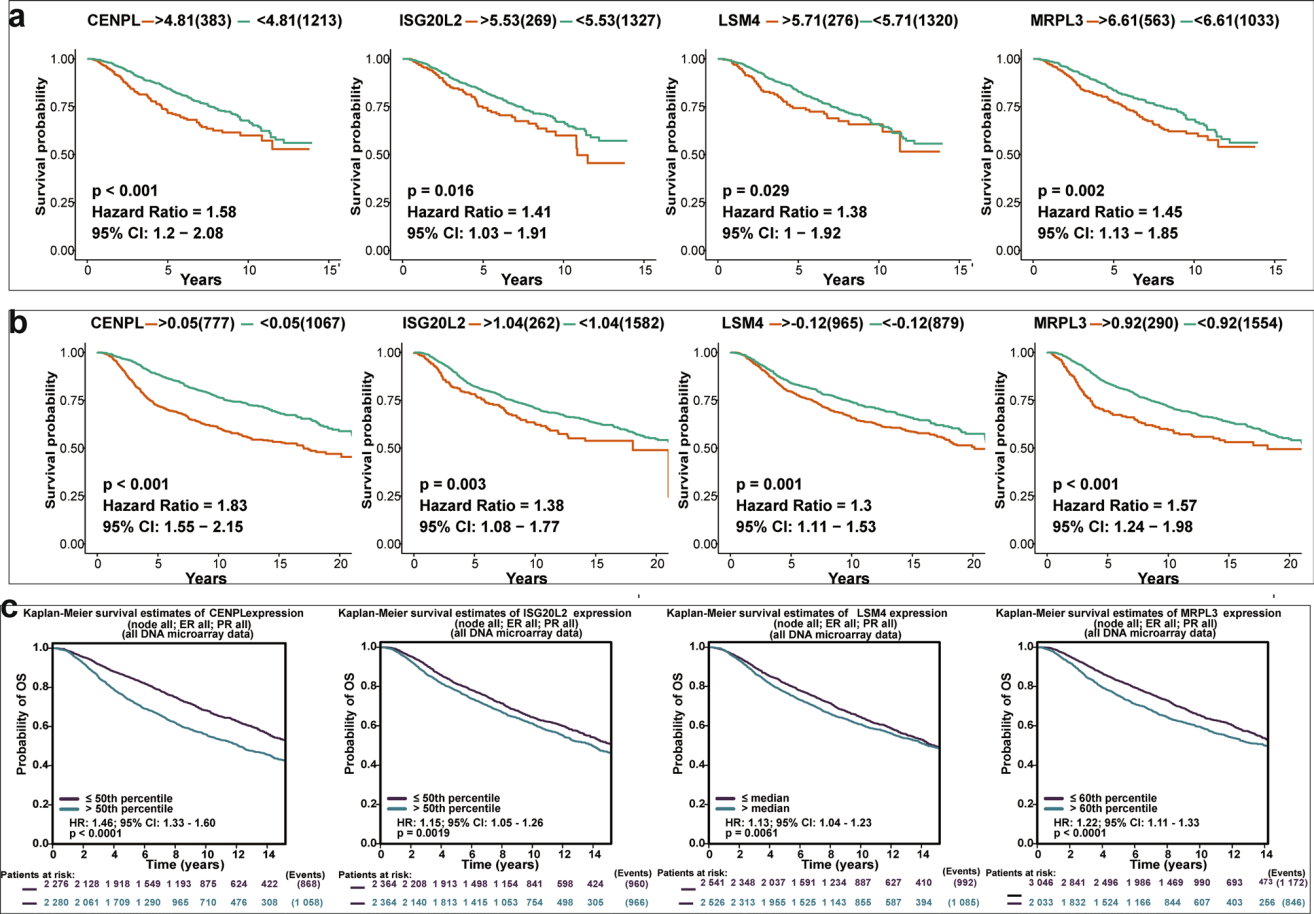
The prognostic value analysis of four novel hub genes in breast cancer based on (**a)** TCGA_GEO BRCA dataset, (**b)** METABRIC dataset, (**c)** bc-GenExMiner v4.6 Platform. Expression levels of CENPL, ISG20L2, LSM4 and MRPL3 are significantly associated with the OS of patients in breast cancer (all P < 0.05, HR˃1).

To estimate the prognostic value of each hub gene in BC patients, we conducted overall survival (OS) analysis using Kaplan–Meier survival method based on three different datasets that contains several thousand breast cancer samples. The Kaplan–Meier curves of each hub gene showed that the difference between high expression groups and low expression groups were significant (all P < 0.05). And the higher expression levels of these four hub genes were significantly associated with the poor OS of breast cancer patients (HR ˃1, Fig. 6).

### GSEA and GSVA exhibit a tight relationship between the four hub genes and tumor cell proliferation

**Figure 7:**
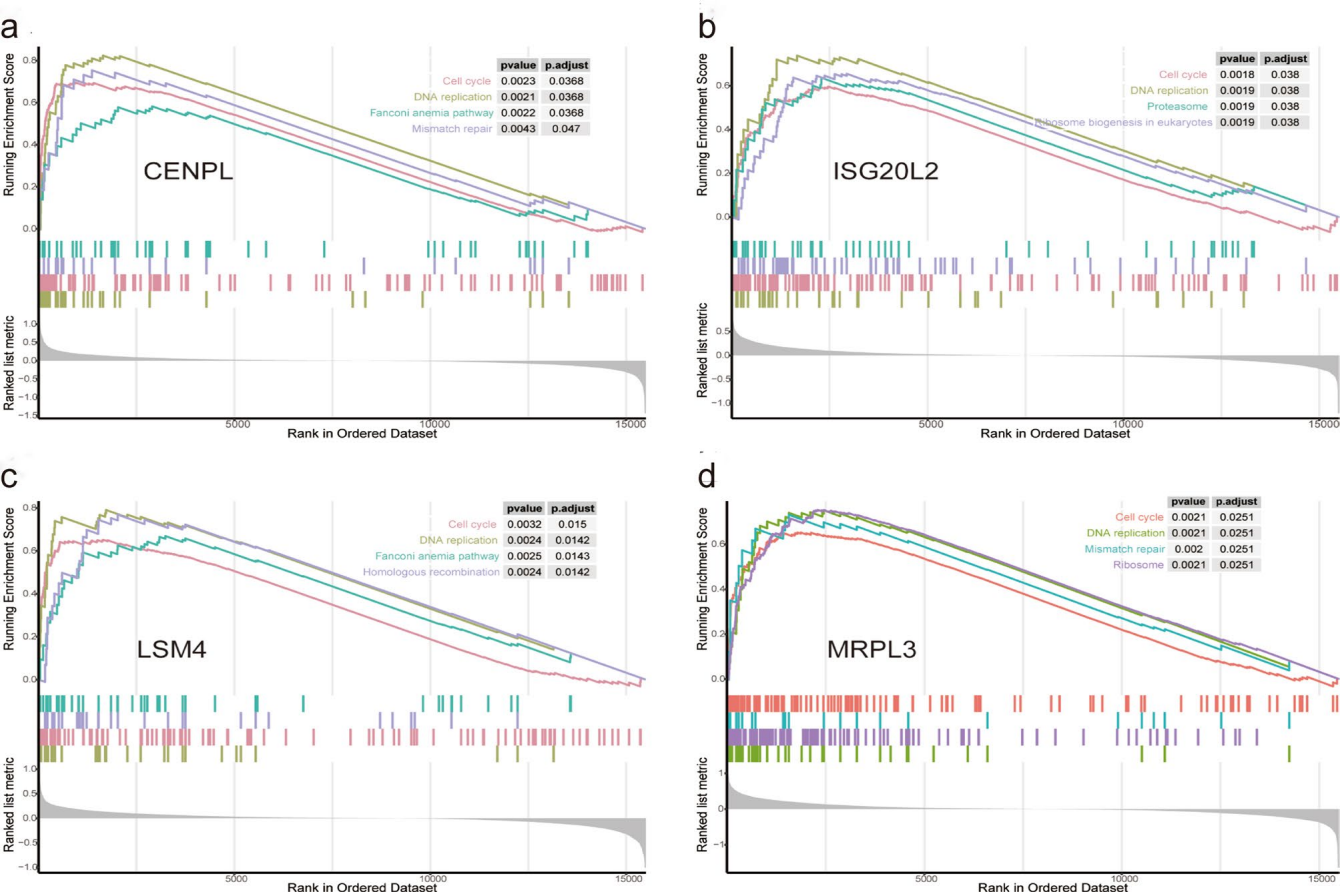
Gene set enrichment analysis (GSEA) of potential hub genes in the METABRC dataset. Tumor cell proliferation related gene-sets were significantly enriched in the high-expression group of each hub gene.

To further elucidate the lurking biological functions of CENPL, ISG20L2, MRPL3 and LSM4 in breast cancer occurrence and development, we conducted GSEA and GSVA using METABRIC dataset. The results of GSEA were shown in Fig. 7, the genes in high expression groups of CENPL, ISG20L2, MRPL3, and LSM4 were all significantly enriched in tumor cell proliferation related pathways such as “cell cycle” and “DNA replication”. Meanwhile, GSVA results substantiated that these cell proliferation-associated gene sets were significantly up-regulated in the high-expression groups of CENPL, ISG20L2, MRPL3 and LSM4 (Supplementary Fig. S9).

### RNA in situ detection

**Figure 8:**
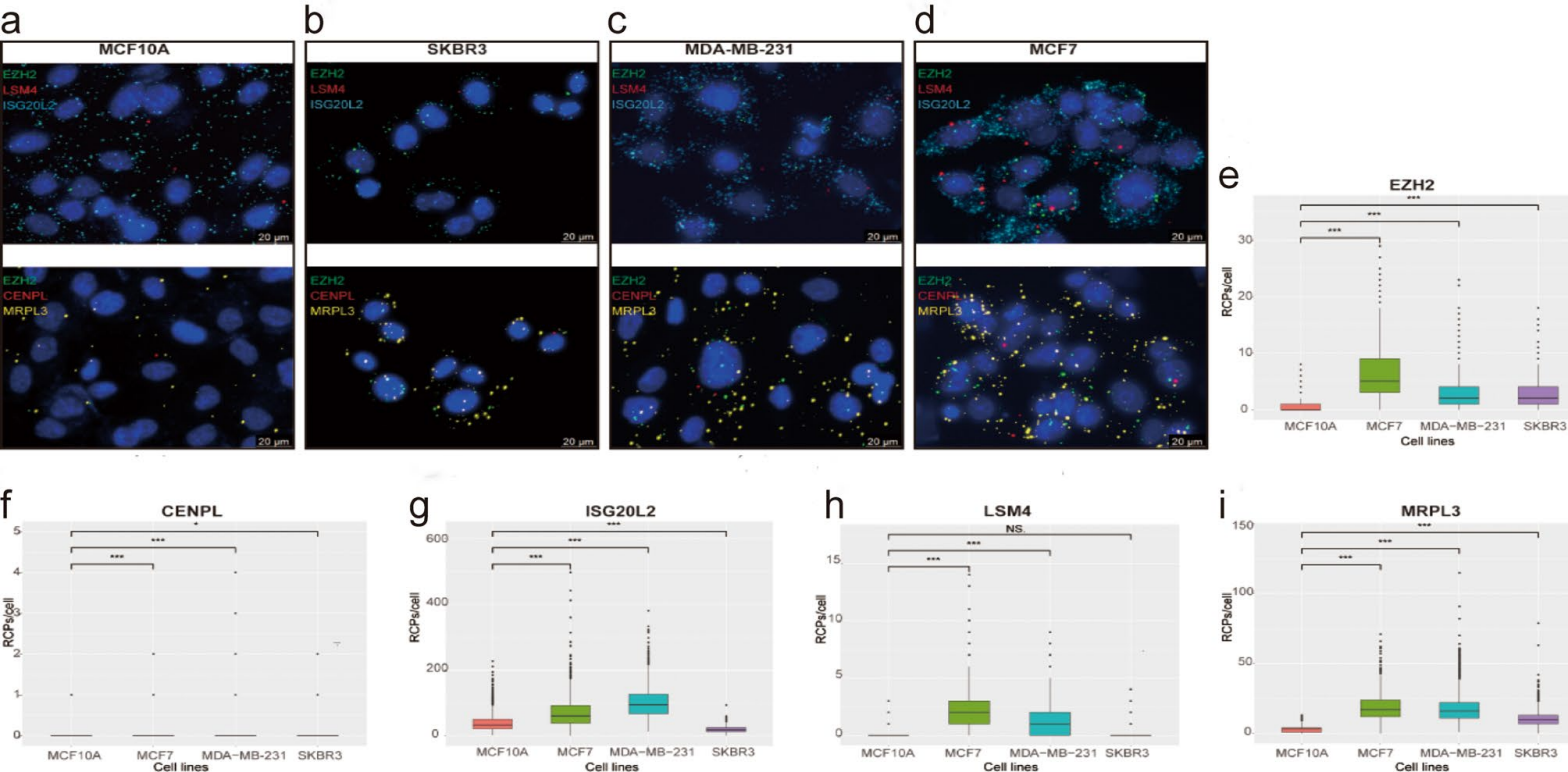
RNA in situ detection of five hub genes in different cell lines. (**a–d)** Demonstration of the expression abundance and spatial localization for each mRNA imaging in single cells. (**a)** five hub genes detection in MCF10A cell; (**b)** five hub genes detection in MDA-MB-231 cell; (**c)** five hub genes detection in MCF7 cell; (**d)** five hub genes detection in SKBR3 cell. (**e–i)** Distribution of RCPs/cell of each probe in four cell lines (MCF10A, MCF7, MDA-MB-231 and SKBR3). NS. Denotes P ˃0.05; * denotes P < 0.05; *** denotes P < 0.001.

We measured the expression abundance and spatial localization of the five hub genes by RNA in situ detection technology. The results indicate that the expression of these five hub genes were mainly distributed in the cytoplasm and nucleus, and the amount of signal originates from hybridization to each probe varied greatly. Among these five genes, ISG20L2 showed the highest expression level, while CENPL and LSM4 have fewer signal (Fig. 8a–d). Compared to normal mammary epithelial cell (MCF10A), the RCPs of each hub gene in both breast cancer cell lines (MCF7 and MDA-MB-231) showed a significant increase (p < 0.05), but the RCPs of ISG20L2 were reduced and the LSM4 were similar as observed in SKBR3 cell (Fig. 8e–i). Considering the five hub genes, which were identified based on PPI networks and WGCNA, share the same signaling pathways during breast cancer progression, we conducted correlation analysis between the four novel hub genes (CENPL, ISG20L2, LSM4 and MRPL3) and EZH2. As shown in Table 4, the expression levels of each hub gene (CENPL, ISG20L2, MRPL3, and LSM4) was correlated with EZH2 in three different breast cancer cell lines (p < 0.01). Furthermore, we also performed the correlations analysis in GEPIA database to assess the four hub genes correlation with EZH2 in breast cancer and the results remained satisfactory (Supplementary Fig. S10, *P* < 0.0001).

**Table 4:** correlation analysis between novel four hub genes (CENPL, ISG20L2, LSM4 and MRPL3) and EZH2 based on RNA in situ detection.

Breast cancer cell lines	Gene	EZH2	*P*
		Cor	
MCF7	CENPL	0.16	4.99e-09
	ISG20L2	0.38	2.34e-46
	LSM4	0.38	1.68e-46
	MRPL3	0.63	2.53e-143
MDA-MB-231	CENPL	0.23	4.64e − 17
	ISG20L2	0.39	1.11e − 46
	LSM4	0.25	2.72e − 18
	MRPL3	0.55	3.84e − 106
SKBR3	CENPL	0.09	9.64e-04
	ISG20L2	0.46	1.85e-62
	LSM4	0.12	3.63e-05
	MRPL3	0.56	4.84e-114

**Cor:** Pearson correlation coefficient.

## Discussion

As a highly prevalent tumor disease worldwide, the complex mechanism involved in the development of breast cancer has not been fully elucidated so far. Thus, identifying potential hub genes involved in breast cancer is not only helpful to elucidating the molecular mechanisms, but also possessing great potentials for the searching of an effective diagnostic biomarkers and prognosis predictors. Previously, hub genes were mainly produced by using a small-scale dataset in most research and showed distressing inconsistent results. In this paper, we applied 10 breast cancer datasets to identify and validate potential novel hub genes so as to guarantee the credibility of the results.

First, 512 robust DEGs between breast tumor tissues and normal breast tissues were identified using RRA method. Functional annotation analysis revealed that the robust DEGs significantly enriched in GO terms were associated with proliferation and energy metabolism, such as extracellular matrix organization, extracellular structure organization, mitotic nuclear division, regulation of lipid metabolic process, and glycosaminoglycan binding, which are implicated in the progression of tumor cell^17–19^. Meanwhile, the KEGG pathway enrichment analysis showed that 22 genes from those robust DEGs were most associated with PI3K—AKT signaling pathway, which serves as a pivotal intracellular signaling path that plays a crucial role in cell cycle regulation and thus involved in breast cancer development^20^. In addition, we also found that those robust DGEs were significantly enriched in PPAR signaling pathway, EC—receptor interaction, AMPK signaling pathway, and multiple studies have shown these pathways activation are participated in the development and progression of breast cancer and affect the final outcomes^21–23^. Based on the PPI networks analysis of robust DEGs, EZH2 was found to be a key gene in the development of breast cancer, and this has been shown in various breast cancer-related studies^24,25^.

Subsequently, a total of 104 hub genes associated with breast cancer were found using the “WGCNA” approach. Among those 104 hub genes, we chose four significantly up-regulated genes, including CENPL, ISG20L2, MRPL3, and LSM4 as hub genes of interest due to the reason that they have rarely been studied in breast cancer and closely related to clinicopathological variables of BC patients. Centromere Protein L (CENPL) is a component of the CENPA-CAD (nucleosome distal) complex, which participated in the assembly process of kinetochore proteins, mitotic progression and chromosome segregation^26^. Interferon stimulated exonuclease gene 20 Like 2 (ISG20L2) encodes a 3'–5' exoribonuclease that involved in the 12S pre-rRNA processing, as a target gene of miR-139-3p, has been reported to take part in the pathogenesis of hepatocellular carcinoma^27,28^. Mitochondrial ribosomal protein L3 (MRPL3), which belongs to the L3P ribosomal protein family, encodes a 39S subunit protein, and plays a regulatory role in the process of Combined Oxidative Phosphorylation^29^. Small nuclear ribonucleoprotein Sm-like4 (LSM4) encodes a member of the LSM family of RNA-binding proteins, has an important role in pre-mRNA splicing by mediating U4/U6 snRNP formation, and this gene has been reported involved in the pathogenesis of pancreatic cancer^30,31^. Interestingly, Joseph S. Baxter et al^32^ have also identified that LSM4 is one of 110 target genes at 33 breast cancer risk loci based on Capture Hi-C technology, which supports the conclusions of the present study. In this study, our data not only showed the expression level of CENPL, ISG20L2, LSM4 and MRPL3 were strongly associated with age, lymph node metastasis and higher SBR grade, but also significantly upregulated in triple negative breast cancer patients compared to non-triple negative breast cancer. Furthermore, they are also correlated with the expression of the tumor cell proliferative marker MKI67 (Ki-67), indicating that these four genes may play vital roles in the pathogenesis and progression of breast cancer.

Next, ROC analysis revealed that the mRNA expression levels of these four hub genes had excellent diagnostic performance for breast cancer. Prognosis analysis showed that these hub genes were high risk genes, and the higher expression levels were related to poorer prognosis for breast cancer patients. Early diagnosis and accurate evaluation of prognosis play an important role in improving the prognosis of breast cancer patients. thus, these four hub genes also have potentials to serve as promising candidate diagnostic biomarkers and prognosis predictors for breast cancer.

Alternatively, we have also undertaken a preliminary analysis of up-regulation mechanism of these four hub genes refer to DiseaseMeth 2.0 and cBioPortal Database. In DiseaseMeth 2.0 database, the correlation analysis of DNA methylation patterns of hub genes with mRNA expression revealed that CENPL, MRPL3 and LSM4 were hypomethylated in breast cancer samples compared with adjacent normal ones, while ISG20L2 was hypermethylated in breast cancer sample. Generally speaking, the DNA methylation patterns negatively correlates with mRNA expression, which is consistent with the observed up-regulation of CENPL, MRPL3 and LSM4 in breast cancer, while the latest research suggested DNA hypermethylation can lead to mRNA upregulation^33^. Moreover, in cBioPortal database, we found that the abnormal expression of the above hub genes in breast cancer were associated with genetic alterations. In Summary, we think that the gene regulation complexity and up-regulation mechanism of the four hub genes should be further studied.

In addition, we used GSEA to further explore the biological functions of the four hub genes in breast cancer and the results showed that the high-expression groups of CENPL, ISG20L2, MRPL3 and LSM4, were significantly enriched in pathways related to cell proliferation such as “cell cycle” pathway. The results of GSVA were in accordance with the GSEA results. Cell-cycle dysregulation is one of the hallmarks of cancer and several researches have reported that cell cycle disturbance is the most important mechanism for cancer occurrence and progression^34^. Given that the specific functions of those novel hub genes remain unclear, additional research are required to investigate the underlying molecular mechanisms in breast cancer.

Finally, RNA in situ detection technology was applied to detect the five hub genes obtained based on bioinformatics methods, and the results showed that the expression of those five hub genes are different but the spatial localization are similar. While verifying the expression differences of each gene, the correlation analysis showed that the CENPL, ISG20L2, MRPL3 and LSM4 was correlated with EZH2 expression. Since EZH2 are currently more studied in breast cancer, and the four novel hub genes (CENPL, ISG20L2, MRPL3, LSM4) and EZH2 are involved in similar signaling pathways derive from GSEA results, we speculate that each novel hub gene has correlation with EZH2 expression, which were verified by the RNA in situ detection results. As an oncogene, growing evidence has identified EZH2 was closely related to breast cancer development and progression through multiple molecular mechanisms^35,36^, but no studies are related to the roles of CENPL, ISG20L2, MRPL3 and LSM4 in breast cancer. Thus, these four novel hub genes as aberrant molecules in the maintenance of breast cancer progression, their exact functional mechanisms deserve further in-depth study.

## Methods

### Collection of breast cancer-related gene expression profile datasets

Thirteen different datasets comprising eleven datasets from GEO database, one dataset from TCGA database (TCGA_BRCA dataset) and one METABRIC dataset, thousands of breast cancer samples and 280 normal breast tissues samples, were included in our study. The eight series matrix files and corresponding platform annotation information files in RRA analyses were downloaded from Gene Expression Omnibus (GEO) database (http://www.ncbi.nlm.nih.gov/geo/), and processed using R package “GEOquery”^37^. The RNA sequencing data normalized by FPKM method, which contains 1066 breast cancer samples and 112 adjacent normal breast tissues samples, were downloaded from The Cancer Genome Atlas (TCGA) data portal (https://portal.gdc.cancer.gov/) (up to May 01, 2020). At the same time, survival time and vital status of each breast cancer sample in TCGA_BRCA dataset were also extracted and used for subsequent overall survival (OS) analysis. The mRNA expression data and clinicopathological characteristics of 1,904 breast cancer samples in METABRIC dataset were acquired from the cBioPortal website (https://www.cbioportal.org/), of which the mRNA expression levels were determined by Illumina Human v3 microarray and normalized by logarithm^38^. In addition, the raw data of three breast cancer related datasets (GSE21422, GSE42568, and GSE65194) derived from the same microarray platform (GPL570 Affymetrix human genome U133A U133 Plus 2.0 array) were collected separately, then merged and preliminarily cleaned using the “GEOquery” package. The SVA function and Combat function were used to standard and remove the batch effect of three different datasets^39,40^. The merged dataset (GEO_BRCA dataset) was used to validate the diagnostic performance of single hub genes in breast cancer. Furthermore, Breast cancer samples microarray gene expression data from three datasets (GSE20685, GSE20711 and GSE58812) and concomitant follow-up information were also acquired from the Gene Expression Omnibus (GEO) database using “GEOquiry” package and merged with TCGA_BRCA dataset using the “ComBat” function, which served as TCGA_GEO BRCA dataset (including 1596 breast cancer patients) to explore the prognostic value of novel hub genes.

### RRA analysis and identification of robust DEGs

To discern the DEGs between breast cancer and normal breast tissue in each dataset from GEO database. The “limma” package in R was adopted to normalize the gene expression data and conduct differential gene expression analysis^41^. The differentially expressed genes (DEGs) in each dataset were sorted by their fold change value. subsequently, R package “RobustRankAggreg” ^10^was applied to integrate the ranked DEGs of 8 datasets from GEO database so that to find the most important and robust DEGs. Finally, those robust DEGs were determined according to the thresholds: |log2fold change|≥ 1 and *P* < 0.05.

### Pathways and GO function enrichment analyses

To identify the biological functions and pathways of those robust DEGs, Gene Ontology (GO) Function and Kyoto Encyclopedia of Genes and Genomes (KEGG) pathway enrichment analyses were conducted using the “clusterProfiler” R package^42^. The GO terms or KEGG pathways with Adjusted P values less than 0.01 indicated statistical significance. Plus, bubble plots were used for visualizing the top 20 enrichment results of GO terms and KEGG pathways.

### PPI network construction and analysis of modules

To identify the key gene of known functions in breast cancer, 512 robust DEGs were mapped to STRING database (STRING, https://string-db.org/, database version 11.0) to construct a PPI network^43^. Nine topological algorithms in Plug-in CytoHubba^44^, consisting of “MCC”, “MNC”, “Degree”, “BottleNeck”, “EcCentricity”, “Closeness”, “Radiality”, “Betweenness” and “Stress” were selected to identify the hub genes in PPI, and the top 10 genes in each topological algorithm were viewed as most stable key gene in PPI analysis. Moreover, the plug-in Molecular Complex Detection (MCODE) ^45^in Cytoscape software was also applied to analyze and recognize the modules in the PPI network. All parameters of the above analysis procedure used were set at default values.

### WGCNA and potential hub genes identification

To screen potential novel hub genes related to breast cancer, the WGCNA algorithm^46^ was used to construct weighted gene co-expression network and identify gene modules that are highly associated with breast cancer. First, gene expression data of the top 3776 up-regulated DEGs obtained by RRA analysis (according to *P* < 0.05) was extracted from TCGA breast cancer dataset and associated with sample information to construct a sample clustering tree. Second, appropriate soft threshold value (5, scale free R^2^ = 0.97) was selected to convert the correlation matrix into adjacency matrix. Subsequently, the resulting adjacency matrix was further converted to topological overlap matrix (TOM) by the TOM similarity algorithm. Referring to the TOM‐based dissimilarity calculation formula, these 3776 genes were classified into different gene modules marked by different colors. Third, the minimal module size was set as 50 genes and the height cut-off as 0.25 to merge the highly similar gene modules. Meanwhile, the correlation value between each module's module eigengene (ME) and samples information were calculated using Pearson correlation coefficient. The candidate gene modules were identified based on the degree of correlation between the module's ME values and samples traits. Genes with gene significance (GS) value greater than 0.3 and module membership (MM) value greater than 0.6 in candidate modules were defined as hub genes for breast cancer. These genes may have stronger association with the progression and development of breast cancer. Finally, these hub genes were further filtered out based on bioinformatics analyses and literature searches.

### Correlation analysis of each hub gene with Clinicopathological Parameters using Breast Cancer Gene-Expression Miner v4.6 (bc-GenExMiner v4.6) online tool

Bc-GenExMiner v4.6 (http://bcgenex.ico.unicancer.fr) 47is a widely used statistical mining online tool for exploring the "correlation", "expression" and "prognostic" analyses of genes of interest in breast cancer by incorporating published annotated breast cancer transcriptomic data (DNA microarrays [n = 11 359] and RNA-seq [n = 4 712]). In this part of the study, the association of each hub genes expression with clinicopathological features in breast cancer was assessed using the "expression" analysis functionalities in bc-GenExMiner v4.6, and the clinicopathological parameters used in this study mainly contained nodal status (N), Age status, Pathological tumor stage, Scarff Bloom & Richardson grade status (SBR), Ki67 status and Basal-like (PAM50) & triple-negative breast cancer status, etc. A p value less than 0.05 was considered statistically significant.

### Validation of differential expression of novel hub gene

The Gene Expression Profiling Interactive Analysis (GEPIA, http://gepia.cancer-pku.cn/) database and The Human Protein Atlas (HPA; http://www.proteinatlas.org/) database were used to validate the differential expression of each hub gene between breast cancer tissue and normal breast tissue from gene expression and protein levels separately.

### Diagnostic performance analyses

With the aid of R package “pROC”^48^, the receiver operating characteristic (ROC) curves analysis was used to evaluate the diagnostic value of each hub gene using in TCGA_BRCA dataset and GEO_BRCA dataset respectively.

### Prognostic value analyses

To assess the prognostic value of each hub gene, both the samples in METABRIC dataset and TCGA_GEO_BRCA dataset were divided into high-expression group and low-expression group based on each hub gene’s best separation cut-off values. Using built-in “survminer” package and “survival” package in R software, the overall survival (OS) rates were calculated via the Kaplan–Meier method, and the difference in the OS rates between high expression group and low expression group of each hub gene was compared by the log-rank test, *P* < 0.05 was considered as difference significant. In parallel, hazard ratio (HR) value at 95% confidence interval (95% CI) of each hub gene was also calculated. HR greater than 1 suggested that the gene increase the risk of breast cancer, and HR less than 1 indicated that the gene was a beneficial factor for breast cancer. Moreover, the prognostic value of each novel hub gene was further assessed using the "prognostic" analysis functionalities in bc-GenExMiner v4.6 Platform.

### Correlation analysis of methylation level and gene expression of hub genes

The human disease methylation database (DiseaseMeth, version 2.0, http://bioinfo.hrbmu.edu.cn/disease meth/) is a database that integrates massive methylation data from microarray and sequencing results, providing the methylation status annotation information of human diseases^49^. This web database was used to compare the difference of methylation levels of each hub gene between breast cancer and normal breast tissues.

### Association analysis of genetic alteration and gene expression of hub genes

The genetic alteration data for each hub gene in the METABRIC dataset samples at the cBioPortal website (http://www.cbioportal.org/) was used to investigate the correlation of genetic alteration and gene expression in breast cancer.

### Gene set enrichment analysis (GSEA) and gene set variation analysis (GSVA) for single hub genes

To find the potential biological functions of single hub genes in breast cancer, R package “clusterprofiler” was chosen to conduct GSEA using METABRAC dataset. Refer to the split-group approach of OS analysis, the samples were divided into “high-expression group” and “low-expression group” based on each hub gene’s best expression separation cut-off value. Gene differential expression analysis between each hub gene’s “high-expression group” and “low-expression group” was carried out using the “limma” R package. Subsequently, based on the ordered list of all genes according to the logFC value, we performed GSEA using the “clusterProfiler” R package, p. adjust < 0.05 was regarded as statistically significant. Moreover, the GSVA was implemented to verify the differential KEGG pathways of high-expression group and low-expression group via R package “GSVA”^50^. The reference gene set “c2.cp.kegg. v7.0. symbols” were obtained from the Molecular Signature Database (MSigDB,

http://software.broadinstitute.org/gsea/msigdb/index.jsp). Cutoff value of differential KEGG pathways was set |logFC|˃0.2, and P < 0.01 was regarded as statistically significant.

### RNA in situ detection technology and image analysis

RNA in situ detection technology was used to determine each hub gene expression at the cellular level. Specific operations were performed referring to literature reported by Ruijie Deng et al^51^ and the main steps include: Design of padlock probe complementary to the target RNA, after the padlock probe hybridize to its target and the padlock probe is connected into a ring through specific Splint R DNA ligase, the rolling-circle amplification (RCA) is initiated under the action of primer and Phi29 DNA polymerase, and Finally, fluorescently labeled probes were added to achieve signal detection. For high detection efficiency, three padlock probes were designed for each hub gene in this study (Supplementary Materials: The padlock probe designed for five hub genes). The cell lines used in this study include: MCF10A, MCF7, MD-MB-231 and SKBR3. Image analysis and quantification of signal intensity from each probe was performed in CellProfiler software. A minimum of 1000 cells was counted for each cell line probe set, and wilcox—test was conducted to compare the rolling circle products (RCPs) of each hub genes between human breast epithelial cell line (MCF10A) and breast cancer cell lines (MCF7, MD-MB-231 or SKBR3). The expression correlation of these hub genes in breast cancer cell lines were analyzed with pearson method. P-value less than 0.05 was considered statistically significant.

### Ethical concerns

Not applicable.

## Supplementary Information

Supplementary Information 1.

Supplementary Information 2.

Supplementary Information 3.

Supplementary Information 4.

Supplementary Information 5.

Supplementary Information 6.

Supplementary Information 7.

Supplementary Information 8.

Supplementary Information 9.

Supplementary Information 10.

Supplementary Information 11.

Supplementary Information 12.

Supplementary Information 13.

Supplementary Information 14.

## Data Availability

Gene expression microarray datasets were downloaded from Gene Expression Omnibus (GEO) database (http://www.ncbi.nlm.nih.gov/geo/). RNA-seq data and corresponding clinical of TCGA_BRCA was acquired from The Cancer Genome Atlas (TCGA) data portal (https://portal.gdc.cancer.gov/) (up to May 01, 2020). The mRNA expression data and clinicopathological characteristics of METABRIC dataset was obtained from the cBioPortal website (https://www.cbioportal.org/).

## References

[1] DeSantis CE (2019). Breast cancer statistics, 2019. CA Cancer J. Clin..

[2] Siegel RL, Miller KD, Jemal A (2019). Cancer statistics, 2019. CA Cancer J. Clin..

[3] Tran VDT (2019). Condition-specific series of metabolic sub-networks and its application for gene set enrichment analysis. Bioinformatics.

[4] Tremblay BL, Guenard F, Lamarche B, Perusse L, Vohl MC (2019). Network analysis of the potential role of DNA methylation in the relationship between plasma carotenoids and lipid profile. Nutrients.

[5] Niemira M (2019). Molecular signature of subtypes of non-small-cell lung cancer by large-scale transcriptional profiling: identification of key modules and genes by weighted gene co-expression network analysis (WGCNA). Cancers (Basel)..

[6] Clarke C (2013). Correlating transcriptional networks to breast cancer survival: a large-scale coexpression analysis. Carcinogenesis.

[7] Zhang B, Horvath S (2005). A general framework for weighted gene co-expression network analysis. Stat. Appl. Genet. Mol. Biol..

[8] Riddell EA, Roback EY, Wells CE, Zamudio KR, Sears MW (2019). Thermal cues drive plasticity of desiccation resistance in montane salamanders with implications for climate change. Nat. Commun..

[9] Chinchilla B, Encinas P, Coll JM, Gomez-Casado E (2020). Differential immune transcriptome and modulated signalling pathways in rainbow trout infected with viral Haemorrhagic Septicaemia virus (VHSV) and its derivative Non-Virion (NV) gene deleted. Vaccines (Basel)..

[10] Kolde R, Laur S, Adler P, Vilo J (2012). Robust rank aggregation for gene list integration and meta-analysis. Bioinformatics.

[11] Xie S (2019). Identification of significant gene and pathways involved in HBV-related hepatocellular carcinoma by bioinformatics analysis. PeerJ.

[12] Sun G (2019). Identification of differentially expressed genes and biological characteristics of colorectal cancer by integrated bioinformatics analysis. J. Cell Physiol..

[13] Liao Y (2019). Identification of candidate genes associated with the pathogenesis of small cell lung cancer via integrated bioinformatics analysis. Oncol. Lett..

[14] Liu L (2019). Identification of key genes and pathways of thyroid cancer by integrated bioinformatics analysis. J. Cell Physiol..

[15] Bedard PL, Hansen AR, Ratain MJ, Siu LL (2013). Tumour heterogeneity in the clinic. Nature.

[16] Narayanan R, Oates AC (2019). Detection of mRNA by whole mount in situ hybridization and DNA extraction for genotyping of Zebrafish embryos. Bio-Protoc..

[17] Wershof E (2019). Matrix feedback enables diverse higher-order patterning of the extracellular matrix. PLoS Comput. Biol..

[18] Jamshidi N, Diehn M, Bredel M, Kuo MD (2014). Illuminating radiogenomic characteristics of glioblastoma multiforme through integration of MR imaging, messenger RNA expression, and DNA copy number variation. Radiology.

[19] Ma J (2020). Alter between gut bacteria and blood metabolites and the anti-tumor effects of Faecalibacterium prausnitzii in breast cancer. BMC Microbiol..

[20] Morgensztern D, McLeod HL (2005). PI3K/Akt/mTOR pathway as a target for cancer therapy. Anticancer Drugs.

[21] Gkretsi V, Stylianou A, Louca M, Stylianopoulos T (2017). Identification of Ras suppressor-1 (RSU-1) as a potential breast cancer metastasis biomarker using a three-dimensional in vitro approach. Oncotarget.

[22] Zhang J (2017). Metformin inhibits tumorigenesis and tumor growth of breast cancer cells by upregulating mir-200c but downregulating AKT2 expression. J. Cancer.

[23] Cheng H (2013). Skp2 regulates subcellular localization of PPARgamma by MEK signaling pathways in human breast cancer. Int. J. Mol. Sci..

[24] Kleer CG (2003). EZH2 is a marker of aggressive breast cancer and promotes neoplastic transformation of breast epithelial cells. Proc. Natl. Acad. Sci. USA.

[25] Li Z (2017). The degradation of EZH2 mediated by lncRNA ANCR attenuated the invasion and metastasis of breast cancer. Cell Death Differ..

[26] Kumar A, Rajendran V, Sethumadhavan R, Purohit R (2013). Identifying novel oncogenes: a machine learning approach. Interdiscip. Sci.

[27] Coute Y (2008). ISG20L2, a novel vertebrate nucleolar exoribonuclease involved in ribosome biogenesis. Mol. Cell Proteomics.

[28] Zhu Y, Zhou C, He Q (2019). High miR-139-3p expression predicts a better prognosis for hepatocellular carcinoma: a pooled analysis. J. Int. Med. Res..

[29] Cahill LS (2020). Structural Variant in Mitochondrial-Associated Gene (MRPL3) induces adult-onset neurodegeneration with memory impairment in the mouse. J. Neurosci..

[30] Gandini R (2008). LSm4 associates with the plasma membrane and acts as a co-factor in cell volume regulation. Cell Physiol. Biochem..

[31] Xue R (2018). Derivation and validation of the potential core genes in pancreatic cancer for tumor-stroma crosstalk. Biomed. Res. Int..

[32] Baxter JS (2018). Capture Hi–C identifies putative target genes at 33 breast cancer risk loci. Nat. Commun..

[33] Harris CJ (2018). A DNA methylation reader complex that enhances gene transcription. Science.

[34] Stewart ZA, Westfall MD, Pietenpol JA (2003). Cell-cycle dysregulation and anticancer therapy. Trends Pharmacol. Sci..

[35] Yamagishi M, Uchimaru K (2017). Targeting EZH2 in cancer therapy. Curr. Opin. Oncol..

[36] Kim KH, Roberts CW (2016). Targeting EZH2 in cancer. Nat. Med..

[37] Davis S, Meltzer PS (2007). GEOquery: a bridge between the Gene Expression Omnibus (GEO) and BioConductor. Bioinformatics.

[38] Curtis C (2012). The genomic and transcriptomic architecture of 2,000 breast tumours reveals novel subgroups. Nature.

[39] Leek JT, Johnson WE, Parker HS, Jaffe AE, Storey JD (2012). The sva package for removing batch effects and other unwanted variation in high-throughput experiments. Bioinformatics.

[40] Irizarry RA (2003). Exploration, normalization, and summaries of high density oligonucleotide array probe level data. Biostatistics.

[41] Ritchie ME (2015). limma powers differential expression analyses for RNA-sequencing and microarray studies. Nucleic Acids Res..

[42] Yu G, Wang LG, Han Y, He QY (2012). clusterProfiler: an R package for comparing biological themes among gene clusters. OMICS.

[43] Szklarczyk D (2019). STRING v11: protein-protein association networks with increased coverage, supporting functional discovery in genome-wide experimental datasets. Nucleic Acids Res..

[44] Chin CH (2014). cytoHubba: identifying hub objects and sub-networks from complex interactome. BMC Syst. Biol..

[45] Bandettini WP (2012). MultiContrast Delayed Enhancement (MCODE) improves detection of subendocardial myocardial infarction by late gadolinium enhancement cardiovascular magnetic resonance: a clinical validation study. J. Cardiovasc. Magn. Reson..

[46] Langfelder P, Horvath S (2008). WGCNA: an R package for weighted correlation network analysis. BMC Bioinformatics.

[47] Jézéquel P, Campone M, Gouraud W (2012). bc-GenExMiner: an easy-to-use online platform for gene prognostic analyses in breast cancer. Breast Cancer Res. Treat..

[48] Robin X (2011). pROC: an open-source package for R and S+ to analyze and compare ROC curves. BMC Bioinform..

[49] Xiong Y (2017). DiseaseMeth version 2.0: a major expansion and update of the human disease methylation database. Nucleic Acids Res..

[50] Hanzelmann S, Castelo R, Guinney J (2013). GSVA: gene set variation analysis for microarray and RNA-seq data. BMC Bioinformatics.

[51] Deng R, Zhang K, Sun Y, Ren X, Li J (2017). Highly specific imaging of mRNA in single cells by target RNA-initiated rolling circle amplification. Chem. Sci..

