# Supplementary material for: CENPL, ISG20L2, LSM4, MRPL3 are four novel hub genes and may serve as diagnostic and prognostic markers in breast cancer
Source: Sci Rep. 2021 Aug 2;11:15610. doi: 10.1038/s41598-021-95068-6 (PMC8328991; doi:10.1038/s41598-021-95068-6)
Supplement: Supplementary file 2 — Supplementary Information 2. [file 41598_2021_95068_MOESM2_ESM.docx]

# Homo sapiens centromere protein L (CENPL), transcript variant 1, mRNA

NCBI Reference Sequence: NM_001127181.2

[GenBank](https://www.ncbi.nlm.nih.gov/nuccore/NM_001127181.2?report=genbank) [Graphics](https://www.ncbi.nlm.nih.gov/nuccore/NM_001127181.2?report=graph)

>NM_001127181.2 Homo sapiens centromere protein L (CENPL), transcript variant 1, mRNA

AGGAATTCAACGAATCAGTGGTGAGGTGCCGCCGCCATGCCTACTACGGGCGGAAATTTGAGAGAAAGCTTCGCACCGGTAGGAAAACTGAGGAGCAACCGAGCACGATTGGAAAGGTTGACGATAGTGGTTAAGTGGCTCGGCCTTGCCCTCAGTTAAAATGGCAATTTTAGTTGCTTCACTGATACGGAATCGGCGAGGCGCCAAGGAGGCTTCCTGCGTGGGGCCGCAGAGCGAGTCGGGAAACGATTTTAAACTGAAGAGGCGGCGGAGGGCCGAATTCCCTTTTCTCAACGGCTTGATTTCAGAGCTGGGCTGGTCTCTGACAGGCTCAGCTGGAGAGGGACGGGTTGGGACGCACTGTCCTTTTGCCCTTCCCCCTCCGCGAGCAGAAGCTGACTCCGCAGGAGCGAGGGTCGCAGAGCTGGGTGAGCGGAAATGTCCCTCCCAGAGTGAAGTCGCAGGGCCCGCCCCGCGTCTGAGGGAGCGGAGGTCTTCCTGGGGGATTTCAGTCTCCACATAGTTTTGGAGCCGGACTTTTGAAGAATGATTCGTGAATCCGGAATGGGTGACAGCGTCATCACGGCATTTTATTGACAGACCATGGATTCTTACAGTGCACCAGAGTCAACTCCTAGTGCATCCTCAAGACCTGAAGATTACTTTATAGGTGCCACTCCTCTGCAGAAACGATTAGAATCGGTCAGGAAGCAGAGTTCATTTATCCTGACTCCACCTCGAAGGAAAATTCCCCAGTGTTCGCAGTTGCAGGAAGATGTTGACCCTCAAAAGGTTGCATTCCTTCTGCATAAACAGTGGACTTTATATAGTTTAACTCCCTTATATAAATTCTCCTATAGTAATCTCAAAGAGTATTCTAGACTTCTCAATGCTTTTATTGTTGCTGAAAAGCAAAAAGGACTTGCTGTGGAAGTGGGAGAAGACTTCAACATCAAAGTGATTTTTTCTACTCTCCTAGGAATGAAAGGAACACAAAGGGACCCGGAAGCATTTCTTGTCCAGGGTCTCATTTTGTCACCCAGGCTGGAGTACAGTGGCACGATCTTGGTTGACTGCAACCTCTGTCTCCTGGGCTCAAGTGATCCTTCCACCTTAGCCTTCCAAGTAGCTGGGACTGCAGGTGCATGCCACCACACTCGGATTGTGTCAAAATCTCAATTGCCATCTGAGAATAGAGAAGGTAAAGTGCTGTGGACTGGCTGGTTCTGCTGTGTATTTGGAGACAGTCTTCTGGAGACTGTTTCAGAAGATTTCACCTGTCTGCCCTTATTCCTTGCAAATGGAGCAGAGTCTAACACAGCAATAATTGGAACTTGGTTTCAGAAAACCTTTGACTGTTATTTCAGTCCTTTAGCAATCAATGCATTTAATCTTTCCTGGATGGCTGCCATGTGGACTGCATGCAAAATGGACCATTATGTGGCTACTACTGAATTTCTTTGGTCTGTACCCTGTAGCCCTCAAAGTCTGGACATTTCTTTCGCAATACATCCAGAGGATGCAAAAGCTCTATGGGACAGTGTCCACAAAACACCTGGGGAGGTTACCCAGGAAGAAGTTGACCTATTCATGGATTGCCTTTATTCACATTTCCATAGACATTTCAAAATTCATTTATCAGCCACAAGATTAGTTCGTGTTTCAACATCTGTAGCTTCAGCACATACTGATGGAAAAATAAAGATTCTGTGTCATAAATACCTTATTGGAGTGTTAGCATATTTGACAGAACTGGCAATTTTTCAAATTGAGTGAAGCCTTATGTGGACTATAAGTTATAGATTATATACTCTTATTGATAACTTGCCTAATTGCTATGCTGAAAGAGACTGCAGGAGAAATAGGCATCTATCTCTGCATCTGTTTTCCCCACCATGCCTTTGGAGTTGCCAAGATGGAAGCCAAGAAGGATCTAGAAGAACAAAGAATATGGTAGTAGATGAGCCACAGCCAGGTGCCCATGTACTAATCATGATAACCTGACATGCCATTCTCAAAATGCTGAGTTGTTAATTTCTTGTCATCTTTAAATATATATATATAGGCTGGGCTTGGTGGCTCACACCTGTAATTCCAGCACTTTGGGAGGCTGAGGTGGGTGGATCATTTGAGGCCAGGAATTCAAGACCAGCCTGGCCAACATGGTGAAACCCCTTCTCTACTGAAAATACAATAATTAGCTGGGCGTGGTGGCACATGCCTATGATCCCAGCTACTGGGGAGGCTGAGGCAGGAGAATCGTTTTAACCCAGAAGACAGGCTGCAGTGAGCCAAGACTGCACCACTGCACTCCAGCCTGGGCAACAAAGTGAGACTCTGCCTCAAAAAATAAAAAAGAAATAAAATAAATAAATACAGCGTTTATTATGTTTAAACTCTTAGGATTCAAATGAGATGTATCTAATGGATATTTCTGATTTGGGTCTTTAAAACCTTTAATCTCTTTTAAGCATTTTAGACTTGCATTCAGAAAGAATTCTGTCTTCTAAAGACATTTTCCTAGCATTTCACTGCGGAGAACTGGCAATCAAAATCCCCTCAAAAGGGAACTAAAGATTAATATACACATTTTTTTAGTCATTAATATGCTTCTGTTTTAAATACAGTTCAACTCCTGTTGCAATGTTGAATTAAATGTTTATTTTAAAATGAAAAAAAAAAAAAAAAAAAAA

mRNA1:CGCCATGCCTACTACGGGCGGAAATTTGAGAG

cDNA1:CTCTCAAATTTCCGCCCGTAGTAGGCATGGCG

mRNA2:GTCTCCACATAGTTTTGGAGCCGGACTTTTGA

cDNA2:TCAAAAGTCCGGCTCCAAAACTATGTGGAGAC

mRNA3:AAGATTTCACCTGTCTGCCCTTATTCCTTGCA

cDNA3:TGCAAGGAATAAGGGCAGACAGGTGAAATCTT

PLP-hCENPL-1 DP3

CGTAGTAGGCATGGCGTTCCTAGTAATC*CCTCAATGCTGCTGCTGTACTAC*TTCAAAGCTCTCAAATTTCCGCC

PLP-hCENPL-2

AAAACTATGTGGAGACTTCCTAGTAATC*CCTCAATGCTGCTGCTGTACTAC*TTCAAAGTCAAAAGTCCGGCTCC

PLP-hCENPL-3

AGACAGGTGAAATCTTTTCCTAGTAATC*CCTCAATGCTGCTGCTGTACTAC*TTCAAAGTGCAAGGAATAAGGGC

# Homo sapiens enhancer of zeste 2 polycomb repressive complex 2 subunit (EZH2), transcript variant 1, mRNA

NCBI Reference Sequence: NM_004456.5

[GenBank](https://www.ncbi.nlm.nih.gov/nuccore/NM_004456.5?report=genbank) [Graphics](https://www.ncbi.nlm.nih.gov/nuccore/NM_004456.5?report=graph)

>NM_004456.5 Homo sapiens enhancer of zeste 2 polycomb repressive complex 2 subunit (EZH2), transcript variant 1, mRNA

GTTTGGCGCTCGGTCCGGTCGCGTCCGACACCCGGTGGGACTCAGAAGGCAGTGGAGCCCCGGCGGCGGCGGCGGCGGCGCGCGGGGGCGACGCGCGGGAACAACGCGAGTCGGCGCGCGGGACGAAGAATAATCATGGGCCAGACTGGGAAGAAATCTGAGAAGGGACCAGTTTGTTGGCGGAAGCGTGTAAAATCAGAGTACATGCGACTGAGACAGCTCAAGAGGTTCAGACGAGCTGATGAAGTAAAGAGTATGTTTAGTTCCAATCGTCAGAAAATTTTGGAAAGAACGGAAATCTTAAACCAAGAATGGAAACAGCGAAGGATACAGCCTGTGCACATCCTGACTTCTGTGAGCTCATTGCGCGGGACTAGGGAGTGTTCGGTGACCAGTGACTTGGATTTTCCAACACAAGTCATCCCATTAAAGACTCTGAATGCAGTTGCTTCAGTACCCATAATGTATTCTTGGTCTCCCCTACAGCAGAATTTTATGGTGGAAGATGAAACTGTTTTACATAACATTCCTTATATGGGAGATGAAGTTTTAGATCAGGATGGTACTTTCATTGAAGAACTAATAAAAAATTATGATGGGAAAGTACACGGGGATAGAGAATGTGGGTTTATAAATGATGAAATTTTTGTGGAGTTGGTGAATGCCCTTGGTCAATATAATGATGATGACGATGATGATGATGGAGACGATCCTGAAGAAAGAGAAGAAAAGCAGAAAGATCTGGAGGATCACCGAGATGATAAAGAAAGCCGCCCACCTCGGAAATTTCCTTCTGATAAAATTTTTGAAGCCATTTCCTCAATGTTTCCAGATAAGGGCACAGCAGAAGAACTAAAGGAAAAATATAAAGAACTCACCGAACAGCAGCTCCCAGGCGCACTTCCTCCTGAATGTACCCCCAACATAGATGGACCAAATGCTAAATCTGTTCAGAGAGAGCAAAGCTTACACTCCTTTCATACGCTTTTCTGTAGGCGATGTTTTAAATATGACTGCTTCCTACATCGTAAGTGCAATTATTCTTTTCATGCAACACCCAACACTTATAAGCGGAAGAACACAGAAACAGCTCTAGACAACAAACCTTGTGGACCACAGTGTTACCAGCATTTGGAGGGAGCAAAGGAGTTTGCTGCTGCTCTCACCGCTGAGCGGATAAAGACCCCACCAAAACGTCCAGGAGGCCGCAGAAGAGGACGGCTTCCCAATAACAGTAGCAGGCCCAGCACCCCCACCATTAATGTGCTGGAATCAAAGGATACAGACAGTGATAGGGAAGCAGGGACTGAAACGGGGGGAGAGAACAATGATAAAGAAGAAGAAGAGAAGAAAGATGAAACTTCGAGCTCCTCTGAAGCAAATTCTCGGTGTCAAACACCAATAAAGATGAAGCCAAATATTGAACCTCCTGAGAATGTGGAGTGGAGTGGTGCTGAAGCCTCAATGTTTAGAGTCCTCATTGGCACTTACTATGACAATTTCTGTGCCATTGCTAGGTTAATTGGGACCAAAACATGTAGACAGGTGTATGAGTTTAGAGTCAAAGAATCTAGCATCATAGCTCCAGCTCCCGCTGAGGATGTGGATACTCCTCCAAGGAAAAAGAAGAGGAAACACCGGTTGTGGGCTGCACACTGCAGAAAGATACAGCTGAAAAAGGACGGCTCCTCTAACCATGTTTACAACTATCAACCCTGTGATCATCCACGGCAGCCTTGTGACAGTTCGTGCCCTTGTGTGATAGCACAAAATTTTTGTGAAAAGTTTTGTCAATGTAGTTCAGAGTGTCAAAACCGCTTTCCGGGATGCCGCTGCAAAGCACAGTGCAACACCAAGCAGTGCCCGTGCTACCTGGCTGTCCGAGAGTGTGACCCTGACCTCTGTCTTACTTGTGGAGCCGCTGACCATTGGGACAGTAAAAATGTGTCCTGCAAGAACTGCAGTATTCAGCGGGGCTCCAAAAAGCATCTATTGCTGGCACCATCTGACGTGGCAGGCTGGGGGATTTTTATCAAAGATCCTGTGCAGAAAAATGAATTCATCTCAGAATACTGTGGAGAGATTATTTCTCAAGATGAAGCTGACAGAAGAGGGAAAGTGTATGATAAATACATGTGCAGCTTTCTGTTCAACTTGAACAATGATTTTGTGGTGGATGCAACCCGCAAGGGTAACAAAATTCGTTTTGCAAATCATTCGGTAAATCCAAACTGCTATGCAAAAGTTATGATGGTTAACGGTGATCACAGGATAGGTATTTTTGCCAAGAGAGCCATCCAGACTGGCGAAGAGCTGTTTTTTGATTACAGATACAGCCAGGCTGATGCCCTGAAGTATGTCGGCATCGAAAGAGAAATGGAAATCCCTTGACATCTGCTACCTCCTCCCCCCTCCTCTGAAACAGCTGCCTTAGCTTCAGGAACCTCGAGTACTGTGGGCAATTTAGAAAAAGAACATGCAGTTTGAAATTCTGAATTTGCAAAGTACTGTAAGAATAATTTATAGTAATGAGTTTAAAAATCAACTTTTTATTGCCTTCTCACCAGCTGCAAAGTGTTTTGTACCAGTGAATTTTTGCAATAATGCAGTATGGTACATTTTTCAACTTTGAATAAAGAATACTTGAACTTGTC

mRNA1:CATACGCTTTTCTGTAGGCGATGTTTTAAATA

cDNA1:TATTTAAAACATCGCCTACAGAAAAGCGTATG

mRNA2:CGGCTCCTCTAACCATGTTTACAACTATCAAC

cDNA2:GTTGATAGTTGTAAACATGGTTAGAGGAGCCG

mRNA3:GGCTGATGCCCTGAAGTATGTCGGCATCGAAA

cDNA3:TTTCGATGCCGACATACTTCAGGGCATCAGCC

PLP-hEZH2-1 DP5

TACAGAAAAGCGTATGTTCCTAGTAATC*AGTAGCCGTGACTATCGACT*TTCAAAGTTATTTAAAACATCGCC

PLP-hEZH2-2

ATGGTTAGAGGAGCCGTTCCTAGTAATC*AGTAGCCGTGACTATCGACT*TTCAAAGTGTTGATAGTTGTAAAC

PLP-hEZH2-3

CTTCAGGGCATCAGCCTTCCTAGTAATC*AGTAGCCGTGACTATCGACT*TTCAAAGTTTTCGATGCCGACATA

# Homo sapiens LSM4 homolog, U6 small nuclear RNA and mRNA degradation associated (LSM4), transcript variant 2, mRNA

NCBI Reference Sequence: NM_001252129.2

[GenBank](https://www.ncbi.nlm.nih.gov/nuccore/NM_001252129.2?report=genbank) [Graphics](https://www.ncbi.nlm.nih.gov/nuccore/NM_001252129.2?report=graph)

>NM_001252129.2 Homo sapiens LSM4 homolog, U6 small nuclear RNA and mRNA degradation associated (LSM4), transcript variant 2, mRNA

AGCGTGTGCAGCGGCGGCGGCGGAAGTGGCCGGCGAGCCCGGTCCCCGCCGGCACCATGTTGGTGGAGCTGAAAAATGGGGAGACGTACAATGGACACCTGGTGAGCTGCGACAACTGGATGAACATTAACCTGCGAGAAGTCATCTGCACGTCCAGGGACGGGGACAAGTTCTGGCGGATGCCCGAGTGCTACATCCGCGGCAGCACCATCAAGTACCTGCGCATCCCCGACGAGATCATCGACATGGTCAAGGAGGAGGTGGTGGCCAAGGGCCGCGGCCGCGGAGGCCTGCAGCAGCAGAAGCAGCAGAAAGGCCGCGGCATGGGCGGCGCTGGCCGAGGTGTGTTTGGTGGCCGGGGCCGAGGTGGGATCCCGGGCACAGGCAGAGGCCAGCCAGAGAAGAAGCCTGGCAGACAGGCGGGCAAACAGTGAGCGCCCACCCAGACCGGCTGCTGCGCCCCCTCCTGCCAGGGTGGCGATTCCGCTCCACAGTCTCGGACGGATCTGCTCAGAAAGGAAGAGGCAGGCGCCAGGGGGAACCCCCTTCGTGTTTTGTGACCCTCCCTTTTAGGTGAAGCCCCTTTTTCTTGCTAAAACCGGCAATTCTCCGGTTAGAAATGTTACTTGGTGTTTTTTGGTTTTGTGAAACGGCCGTCCCAAAACTGGCTGGATTCCTAGAAGAGTCTGTGTTGAAGGCATCTTTCAAGCCCTCGCTCTGGTTCTCAGGGCAGCATTTTCCAGGCGGGTTTGTTTTGCATTTCTTGGAGCCTCTCCGAGCAGCAACCAGACGGGAGATTTTTATTTTAAGCTGTTCATGCTGGGACTGACAGCCTGCAGGGTTTCCTTGGGCGCGGCCCCAAAATTGCCTTCAAAACAAACCCGGGACGGTTGAAAGCCTTCGAACCGTGCAGGGGATGCCTCGGGCCCTGGCCCTTCGCTTCCTCTCTTGTGTTATGGAAATAAAAACAAATAAAACTACACACTGCACATCCACTGTTCCTTTTGAGCTTCTGGGTGCACCTAGCGGGTCTCCTGCCCTCTGTCCATCGCTGGACTCTCAGGAAATCCCCGTTCCCGTAAGGGAAGCTTCCTTTTGAGAGTTCTCACCAGGTGTCAGCGGCACAGTGAGGCACTGATGTCCTTTTACAAAAGGCAAGACCAGCATCTGGACAGTGGGGGCTCTTGAGAGTCCCCGGCGCCCCCCACACCAGGTTGTCCTATAACCCTCTCCCCTCTGTGGAGACGTTAATGCCAAGGGGTGTGTGGGGAGGGAAGTCCCAGGCAGGGGCCACGCATGGTCCCCATCACCCCCTCCTGGGTGGCCTGGACTCCATCATCTATCCTCATCTGTCCACACTGGCGTTCACACAGAGAAGACAGGACAGGAATCCCCTCGGAAGATGCTCATGGAAGATTCTCACGAGGCTCCTGCCCCCTTTCTGAGCCAGGCCGGGTTTGCCACTCCTCGGGCGCGCACCTGAGACACTGAGTCAGCGCTTTCGAGAAAGGCTCCGGGTAGCATCTGTGGACCGTGAAGAAACCAGAGGGAAGCCCTGAGCCAGGCAGCGGCCCGTCCCCCTGCATAGCCTTCTCTTCAGGGTCCTCGTTCGGAGACCTATTTTGAGAGGTGATTGGAATAAAAGGTCCCGTCCGTGAGGAA

mRNA1:GCTGCGACAACTGGATGAACATTAACCTGCGA

cDNA1:TCGCAGGTTAATGTTCATCCAGTTGTCGCAGC

mRNA2:TACTTGGTGTTTTTTGGTTTTGTGAAACGGCC

cDNA2:GGCCGTTTCACAAAACCAAAAAACACCAAGTA

mRNA3:CTGGCGTTCACACAGAGAAGACAGGACAGGAA

cDNA3:TTCCTGTCCTGTCTTCTCTGTGTGAACGCCAG

PLP-hLSM4-1 DP3

ATCCAGTTGTCGCAGCTTCCTAGTAATC*CCTCAATGCTGCTGCTGTACTAC*TTCAAAGTTCGCAGGTTAATGTTC

PLP-hLSM4-2

CAAAAAACACCAAGTATTCCTAGTAATC*CCTCAATGCTGCTGCTGTACTAC*TTCAAAGTGGCCGTTTCACAAAAC

PLP-hLSM4-3

TCTGTGTGAACGCCAGTTCCTAGTAATC*CCTCAATGCTGCTGCTGTACTAC*TTCAAAGTTTCCTGTCCTGTCTTC

# Homo sapiens mitochondrial ribosomal protein L3 (MRPL3), mRNA; nuclear gene for mitochondrial product

NCBI Reference Sequence: NM_007208.4

[GenBank](https://www.ncbi.nlm.nih.gov/nuccore/NM_007208.4?report=genbank) [Graphics](https://www.ncbi.nlm.nih.gov/nuccore/NM_007208.4?report=graph)

>NM_007208.4 Homo sapiens mitochondrial ribosomal protein L3 (MRPL3), mRNA; nuclear gene for mitochondrial product

GCAGAGAGCATCGGCCGGCGACCGTTCCGGCGGCCATTGCGAAAACTTCCCCACGGCTACTGCGTCCACGTGGCGGTGGCGTGGGGACTCCCTGAAAGCAGAGCGGCAGGGCGCCCGGAAGTCGTGAGTCGAGTCTTCCCGGGCTAATCCATGCCGGGTTGGAGGCTGCTGACGCAGGTCGGCGCCCAGGTGCTGGGTCGACTCGGGGACGGCCTGGGTGCTGCCCTGGGCCCGGGGAACAGAACACACATCTGGCTTTTTGTTAGAGGTCTTCATGGAAAGAGTGGTACATGGTGGGATGAGCATCTTTCTGAAGAAAATGTCCCATTCATTAAGCAGTTGGTCTCTGATGAAGATAAAGCCCAATTAGCAAGTAAACTGTGTCCTCTGAAAGATGAACCATGGCCTATACATCCTTGGGAACCAGGTTCCTTTAGAGTTGGTCTTATTGCCTTGAAGCTGGGCATGATGCCTTTATGGACCAAGGATGGTCAAAAGCATGTGGTCACATTACTTCAGGTACAAGACTGTCATGTCTTAAAATATACGTCAAAGGAAAACTGTAATGGAAAAATGGCAACCCTGTCTGTAGGAGGAAAAACTGTATCACGTTTTCGTAAAGCTACATCCATATTGGAATTTTACCGGGAACTTGGATTGCCGCCGAAACAGACAGTTAAAATCTTTAATATAACAGATAATGCTGCAATTAAACCAGGCACTCCTCTTTATGCTGCTCACTTTCGTCCAGGACAGTATGTGGATGTCACAGCCAAAACTATTGGTAAAGGTTTTCAAGGTGTCATGAAAAGATGGGGATTTAAAGGCCAGCCTGCTACGCATGGTCAAACGAAAACCCACAGGAGACCTGGAGCTGTTGCAACTGGTGATATTGGCAGAGTCTGGCCTGGAACTAAAATGCCTGGAAAAATGGGAAACATATACAGGACAGAATATGGACTGAAAGTGTGGAGAATAAACACAAAGCACAACATAATCTATGTAAATGGCTCTGTACCTGGACATAAAAATTGCTTAGTAAAGGTCAAAGATTCTAAACTGCCTGCATATAAGGATCTCGGTAAAAATCTACCATTCCCTACATATTTTCCTGATGGAGATGAAGAGGAACTGCCAGAAGATTTGTATGATGAAAACGTGTGTCAGCCCGGTGCGCCTTCTATTACATTTGCCTAACATCTTTGGACGTGGCAGAACCTTACATATTCTGTGAGCTTCGATGAGCCAGAGTGATATCATAACCACCAGAAATCATACTCTCCTTTCTTAGTCACAACAAAATCACACATGTCATCTTTGTCAAGGGCATAAATATATCATTCATACCCCCATTAAATTTTGTTAGAAAAATTACCACATTAAATATATGAGTTAAGTAGATTGGATTTGCTGAAATTGGTGTTGGGCATATTAGCAAAATATTCTTAATTTGTGGACTCGATTCTTTTTTACTACATATTTCCCAAGTTATCTTAAGATGTCTGTAAATTTAACTTTTATTAAAGTTTTGTCAATCTTTGTGAAATAGTGGTTGTGGAACAGTAGAAAACCATATGGGGACTATAGTGCAACCTATTTGGGTAAAGAAACCATTTGCTAAAATGGAGAAAGTAAATAGATTTTTATTTAAATTACAGAAACATGTTAAAGGCCGGACAAAGGAAAGACAATAAAATCATAAATTATC

mRNA1:TATTGGAATTTTACCGGGAACTTGGATTGCCG

cDNA1:CGGCAATCCAAGTTCCCGGTAAAATTCCAATA

mRNA2:CGAAAACTTCCCCACGGCTACTGCGTCCACGT

cDNA2:ACGTGGACGCAGTAGCCGTGGGGAAGTTTTCG

mRNA3:TTTGCCTAACATCTTTGGACGTGGCAGAACCT

cDNA3:AGGTTCTGCCACGTCCAAAGATGTTAGGCAAA

PLP-hMRPL3-1 DP2

CGGTAAAATTCCAATATTCCTAGTAATC*CCTCAATGCACATGTTTGGCTCC*TTCAAAGTCGGCAATCCAAGTTCC

PLP-hMRPL3-2

CGTGGGGAAGTTTTCGTTCCTAGTAATC*CCTCAATGCACATGTTTGGCTCC*TTCAAAGTCGGCAATCCAAGTTCC

PLP-hMRPL3-3

AAAGATGTTAGGCAAATTCCTAGTAATC*CCTCAATGCACATGTTTGGCTCC*TTCAAAGTAGGTTCTGCCACGTCC

# Homo sapiens interferon stimulated exonuclease gene 20 like 2 (ISG20L2), transcript variant 3, mRNA

NCBI Reference Sequence: NM_001303095.1

[GenBank](https://www.ncbi.nlm.nih.gov/nuccore/NM_001303095.1?report=genbank) [Graphics](https://www.ncbi.nlm.nih.gov/nuccore/NM_001303095.1?report=graph)

>NM_001303095.1 Homo sapiens interferon stimulated exonuclease gene 20 like 2 (ISG20L2), transcript variant 3, mRNA

GGTCTTAGCTGGACCCGTCTGGGAGGTAGGTTTGTGAGCGTGAGAGATCGATCTGTACCGCGGGGATCCGAAGTATGCTTATCCAGGGTGTACTACAGACCTCCACATGTTGGACAGTAGGATTCAGCAGAGAATGAGTCCCACATCCTCTTTTCATCCATAAGCCACTCTTCCCAACTACTCTTCCGCCTTCCATTGTCCCTATGTCTACTTTACTGCTCAATCTGGATTTTGGGGAACCTCCTCCCAAAAAGGCATTAGAAGGAAATGCCAAGCACCGAAATTTTGTCAAGAAGCGGAGGCTCTTAGAACGGAGAGGCTTTCTGAGTAAAAAGAACCAACCCCCTAGCAAGGCGCCTAAGTTGCACTCTGAACCTTCAAAGAAAGGGGAAACTCCTACGGTCGATGGCACTTGGAAGACCCCTTCCTTCCCAAAAAAGAAGACAGCTGCTTCCAGCAATGGGTCAGGACAGCCCCTGGACAAGAAAGCTGCAGTGTCTTGGTTGACCCCTGCCCCTTCAAAAAAGGCTGATTCTGTTGCTGCTAAAGTAGATTTGCTGGGGGAGTTCCAGAGTGCCCTTCCAAAGATCAATAGCCACCCAACCCGCTCTCAGAAGAAGAGCTCCCAGAAGAAATCCTCTAAAAAGAACCATCCTCAGAAGAATGCCCCACAGAACTCCACCCAAGCTCATTCAGAGAATAAATGCTCCGGAGCATCCCAGAAGTTGCCACGGAAGATGGTGGCAATTGACTGTGAGATGGTGGGCACAGGACCAAAGGGGCATGTTAGTTCCTTGGCTCGATGTAGCATTGTCAACTACAACGGAGATGTGCTTTATGACGAGTACATTCTTCCCCCCTGCCACATTGTGGACTACCGAACCAGGTGGAGTGGTATCCGGAAGCAGCACATGGTGAATGCCACACCCTTCAAGATTGCTCGAGGCCAGATCTTGAAGATACTCACAGGGAAGATAGTGGTGGGGCATGCCATCCACAACGACTTCAAAGCCCTTCAGTACTTTCACCCCAAGTCCCTCACCCGTGACACCTCCCATATCCCCCCCCTCAACCGGAAGGCTGACTGCCCGGAGAATGCCACCATGTCTCTGAAGCATCTCACCAAGAAGCTGCTAAACCGGGATATCCAGGTTGGGAAGAGCGGACATTCCTCTGTGGAAGATGCCCAGGCCACCATGGAGCTATATAAGTTGGTTGAAGTCGAGTGGGAAGAGCACCTAGCCCGGAATCCCCCTACAGACTAGTGGCAGTGGGGACGCTGGTGATATGAGGAGGCAGAGGCAGCACCCAGGAGAAACAGGGCAGTGGACCAATGGACAGCTCCACCAGCTCCACATCTTTGGAAGCTAGATTTGGGGAGAGAGAAGCTCTACCCCAGACTTAATACCCATTGAAATTTCACCTCAGGTGTTGTGTCCTGTGTCTGGTTAAGTGTCCCATGGAAGGGGAAAGCCTTCACGTCAGAACCCAACCCTATACCTTTTACTTCTTAAATGGTGCTAACCACAGGTGTCCCAGGGTGCTCTGTGCCAGTTAAGATTTTTAACTTTCAAGGGGCAGGGCATACTGGGAAATGTAGTTTCCCAAACTGCCTTATCACTTGGGTGGACATATGTCTCCTTTTATGCCTTTTGGTCTTGAGTAATTAACAGCATCCTCTTCCACGCTCAGAAGTGTTCTGGTTGGGGCCAGGCATGGTGGCTCACGCCTGTAGTCCCAACACTTAGGGAGGCCGAGGCGGGCGGATCACCTGAGATCAGGAGTTCAAGACCAGCCTGGCCAACATGGCGAATTCCCGTTCTCTACTAAAAATACAAAAAATGTGTGGGGTGTGGTGGCAGGAGCCTGTAATCCTAGCTACTCAGGAGGCTGAGGCAGGAGAATCGCTTGAGCCCAGGAGGCGGAGATTGCAGTGAGCCGAGATCGTGTCACTGCACTCCAGCCTGGGTGACAAGAGTGAGACTCCGTCTCCAAAAAAAAAAAAAAAGAATTTATTAACCTGGTTGGTTAATAAATTGTGTGGTCCCTGCCGGGCGTGGTGGCTCACGCCTGTAATCCCAGCACTTTGGGAGGCCGAGGCGGGCAGATCACGAGGTCAGGAGATCGAGACCATCCTGGCTAACACGGTGAAACCCCGTCTCTACTAAAAATACAAAAAATTAGCCAGGCGTGGTGGCGGGCACCTGTAGTTCCAGCTACTTGGGAGGCTGAGGCAGGAGAATGGCGTGAACCCAGGAGGCGGAGCTTGCAGTGAGCCGAGATTGTGCCACTGCACTCCAGCCTGGGCCACGGCCACAGAGCGAGACTCTGTCTCAAAAAAAAAAAAAAATTAAAATTGTGTGGTCCCTTTCCATGTCACAGAGTCTGCTTTTGGCTCAGAGTCTGTATCTAGCCTGTGTCAGGATATTGCCTGTTCTTCCCACCTTGACTTCCAGAGCAGCCATCGCTGGTGAGCTTTAGTTCTCCTTAGGGTTCCAAGGCATTAGAAGTAAGAGTGTAGCTTGGAAACCAACTTCTGTGATTTAGGAAAGCTGTCTCTTTCCTAAGGTAATAGGGGGTTTATTTCATTTAGGCCCCAGTTGGTAGGTTTTAAAATAATTTATAAAGTGTTTTAATAATTTGGTTGTCTTTTTTACCTTGCTAAACCAAGCCTCTAAAAACTCAAATTATTCCCTTGATAAATTTTTATAATGGAAAAGCAAAAAAAAAAAAAAAAA

mRNA1:AAGTCGAGTGGGAAGAGCACCTAGCCCGGAAT

cDNA1:ATTCCGGGCTAGGTGCTCTTCCCACTCGACTT

mRNA2:ATCGATCTGTACCGCGGGGATCCGAAGTATGC

cDNA2:GCATACTTCGGATCCCCGCGGTACAGATCGAT

mRNA3:CCCACCTTGACTTCCAGAGCAGCCATCGCTGG

cDNA3:CCAGCGATGGCTGCTCTGGAAGTCAAGGTGGG

PLP-hISG20L2-1 DP4

TCTTCCCACTCGACTTTTCCTAGTAATC*TCGCGCTTGGTATAATCGCT*TTCAAAGTATTCCGGGCTAGGTGC

PLP-hISG20L2-2

CGCGGTACAGATCGATTTCCTAGTAATC*TCGCGCTTGGTATAATCGCT*TTCAAAGTGCATACTTCGGATCCC

PLP-hISG20L2-3

TGGAAGTCAAGGTGGGTTCCTAGTAATC*TCGCGCTTGGTATAATCGCT*TTCAAAGTCCAGCGATGGCTGCTC
