# Supplementary material for: CENPL, ISG20L2, LSM4, MRPL3 are four novel hub genes and may serve as diagnostic and prognostic markers in breast cancer
Source: Sci Rep. 2021 Aug 2;11:15610. doi: 10.1038/s41598-021-95068-6 (PMC8328991; doi:10.1038/s41598-021-95068-6)
Supplement: Supplementary file 5 — Supplementary Information 5. [file 41598_2021_95068_MOESM5_ESM.docx]

**Table S3 Top 30 GO enrichment terms and KEGG pathway analysis of genes in blue module**

| **Category** | **ID** | **Description** | **GeneRatio** | **p.adjust** |
| --- | --- | --- | --- | --- |
| BP | GO:0007059 | chromosome segregation | 80/857 | 3.78E-33 |
| BP | GO:0048285 | organelle fission | 94/857 | 4.37E-33 |
| BP | GO:0000280 | nuclear division | 89/857 | 6.79E-33 |
| BP | GO:0140014 | mitotic nuclear division | 68/857 | 2.76E-29 |
| BP | GO:0000070 | mitotic sister chromatid segregation | 52/857 | 8.23E-29 |
| BP | GO:0098813 | nuclear chromosome segregation | 67/857 | 8.62E-29 |
| BP | GO:0000819 | sister chromatid segregation | 56/857 | 1.99E-27 |
| BP | GO:0006260 | DNA replication | 63/857 | 2.85E-24 |
| BP | GO:0000075 | cell cycle checkpoint | 56/857 | 2.97E-24 |
| BP | GO:0006261 | DNA-dependent DNA replication | 46/857 | 1.10E-22 |
| CC | GO:0098687 | chromosomal region | 83/887 | 2.38E-34 |
| CC | GO:0000775 | chromosome, centromeric region | 59/887 | 1.29E-30 |
| CC | GO:0000793 | condensed chromosome | 57/887 | 3.37E-25 |
| CC | GO:0000776 | kinetochore | 45/887 | 3.92E-25 |
| CC | GO:0000779 | condensed chromosome, centromeric region | 42/887 | 8.40E-25 |
| CC | GO:0005819 | spindle | 67/887 | 1.70E-22 |
| CC | GO:0000777 | condensed chromosome kinetochore | 37/887 | 1.01E-21 |
| CC | GO:0000922 | spindle pole | 36/887 | 1.12E-13 |
| CC | GO:0072686 | mitotic spindle | 28/887 | 2.11E-12 |
| CC | GO:0030496 | midbody | 32/887 | 4.66E-10 |
| MF | GO:0140097 | catalytic activity, acting on DNA | 46/859 | 4.91E-15 |
| MF | GO:0003678 | DNA helicase activity | 23/859 | 1.19E-09 |
| MF | GO:0016887 | ATPase activity | 50/859 | 5.12E-08 |
| MF | GO:0003697 | single-stranded DNA binding | 24/859 | 1.53E-07 |
| MF | GO:0004386 | helicase activity | 29/859 | 1.82E-07 |
| MF | GO:0008094 | DNA-dependent ATPase activity | 18/859 | 4.18E-07 |
| MF | GO:0003688 | DNA replication origin binding | 11/859 | 5.32E-07 |
| MF | GO:0070182 | DNA polymerase binding | 9/859 | 8.61E-06 |
| MF | GO:0043138 | 3'-5' DNA helicase activity | 9/859 | 1.33E-05 |
| MF | GO:0008017 | microtubule binding | 32/859 | 2.99E-05 |
| KEGG | hsa04110 | Cell cycle | 38/429 | 3.25E-17 |
| KEGG | hsa03030 | DNA replication | 17/429 | 7.00E-11 |
| KEGG | hsa03440 | Homologous recombination | 12/429 | 7.54E-05 |
| KEGG | hsa04141 | Protein processing in endoplasmic reticulum | 24/429 | 0.000717847 |
| KEGG | hsa04114 | Oocyte meiosis | 19/429 | 0.00245488 |
| KEGG | hsa03460 | Fanconi anemia pathway | 11/429 | 0.004558725 |
| KEGG | hsa05110 | Vibrio cholerae infection | 10/429 | 0.008726129 |
| KEGG | hsa03410 | Base excision repair | 8/429 | 0.008726129 |
| KEGG | hsa03013 | RNA transport | 22/429 | 0.009840956 |
| KEGG | hsa04914 | Progesterone-mediated oocyte maturation | 14/429 | 0.020781174 |
| KEGG | hsa03430 | Mismatch repair | 6/429 | 0.025367537 |
| KEGG | hsa04218 | Cellular senescence | 18/429 | 0.033899541 |

**Table S4** GO enrichment terms and KEGG pathway analysis of genes in brown module

| **Category** | **ID** | **Description** | **GeneRatio** | **p.adjust** |
| --- | --- | --- | --- | --- |
| CC | GO:0101002 | ficolin-1-rich granule | 19/669 | 0.010668439 |
| CC | GO:1904813 | ficolin-1-rich granule lumen | 14/669 | 0.018010486 |
| CC | GO:0016324 | apical plasma membrane | 25/669 | 0.018010486 |
| CC | GO:0033176 | proton-transporting V-type ATPase complex | 6/669 | 0.025574899 |
| CC | GO:0033179 | proton-transporting V-type ATPase, V0 domain | 4/669 | 0.025574899 |
| CC | GO:0016327 | apicolateral plasma membrane | 5/669 | 0.025574899 |
| CC | GO:0045177 | apical part of cell | 27/669 | 0.025790899 |
| CC | GO:0043296 | apical junction complex | 14/669 | 0.026977725 |
| CC | GO:0070160 | tight junction | 13/669 | 0.026977725 |
| CC | GO:0005911 | cell-cell junction | 30/669 | 0.029474562 |
| CC | GO:0005938 | cell cortex | 22/669 | 0.043999282 |
| CC | GO:0005774 | vacuolar membrane | 27/669 | 0.043999282 |
| CC | GO:0005923 | bicellular tight junction | 12/669 | 0.043999282 |
| CC | GO:0098852 | lytic vacuole membrane | 24/669 | 0.046955223 |
| CC | GO:0005925 | focal adhesion | 26/669 | 0.047991329 |
| CC | GO:0044448 | cell cortex part | 15/669 | 0.047991329 |
| CC | GO:0030286 | dynein complex | 7/669 | 0.047991329 |
| CC | GO:0036452 | ESCRT complex | 5/669 | 0.047991329 |
| CC | GO:0005924 | cell-substrate adherens junction | 26/669 | 0.047991329 |
| CC | GO:0071004 | U2-type prespliceosome | 4/669 | 0.047991329 |
| CC | GO:0071010 | prespliceosome | 4/669 | 0.047991329 |
| CC | GO:0030055 | cell-substrate junction | 26/669 | 0.048868953 |
| CC | GO:0036064 | ciliary basal body | 12/669 | 0.048868953 |
| MF | GO:0003697 | single-stranded DNA binding | 24/859 | 1.53E-07 |
| KEGG | hsa04530 | Tight junction | 17/287 | 0.026062904 |
