# Supplementary figures and images for: CENPL, ISG20L2, LSM4, MRPL3 are four novel hub genes and may serve as diagnostic and prognostic markers in breast cancer
Source: Sci Rep. 2021 Aug 2;11:15610. doi: 10.1038/s41598-021-95068-6 (PMC8328991; doi:10.1038/s41598-021-95068-6)

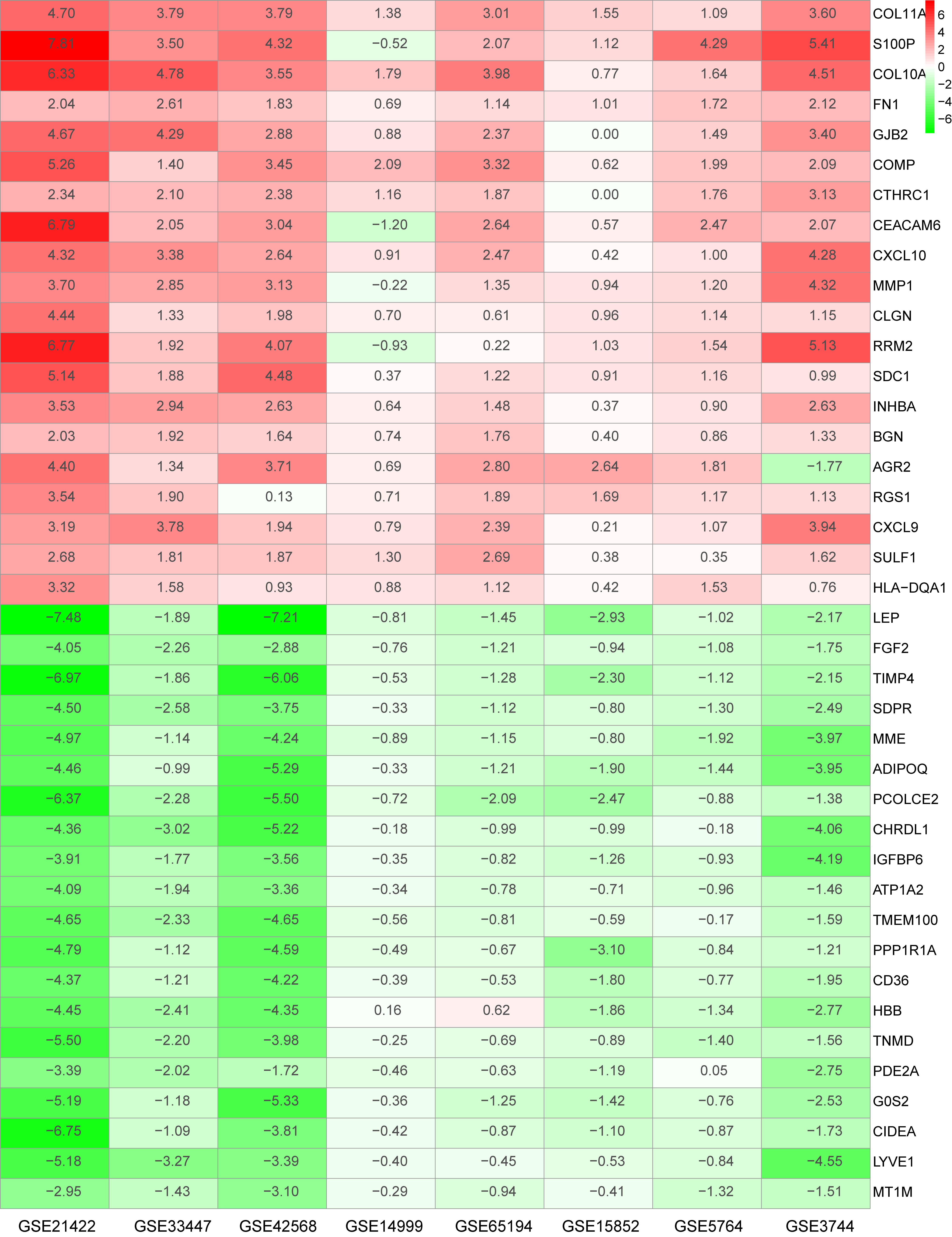

Supplement: Supplementary file 6 — Supplementary Information 6. [file 41598_2021_95068_MOESM6_ESM.tif]

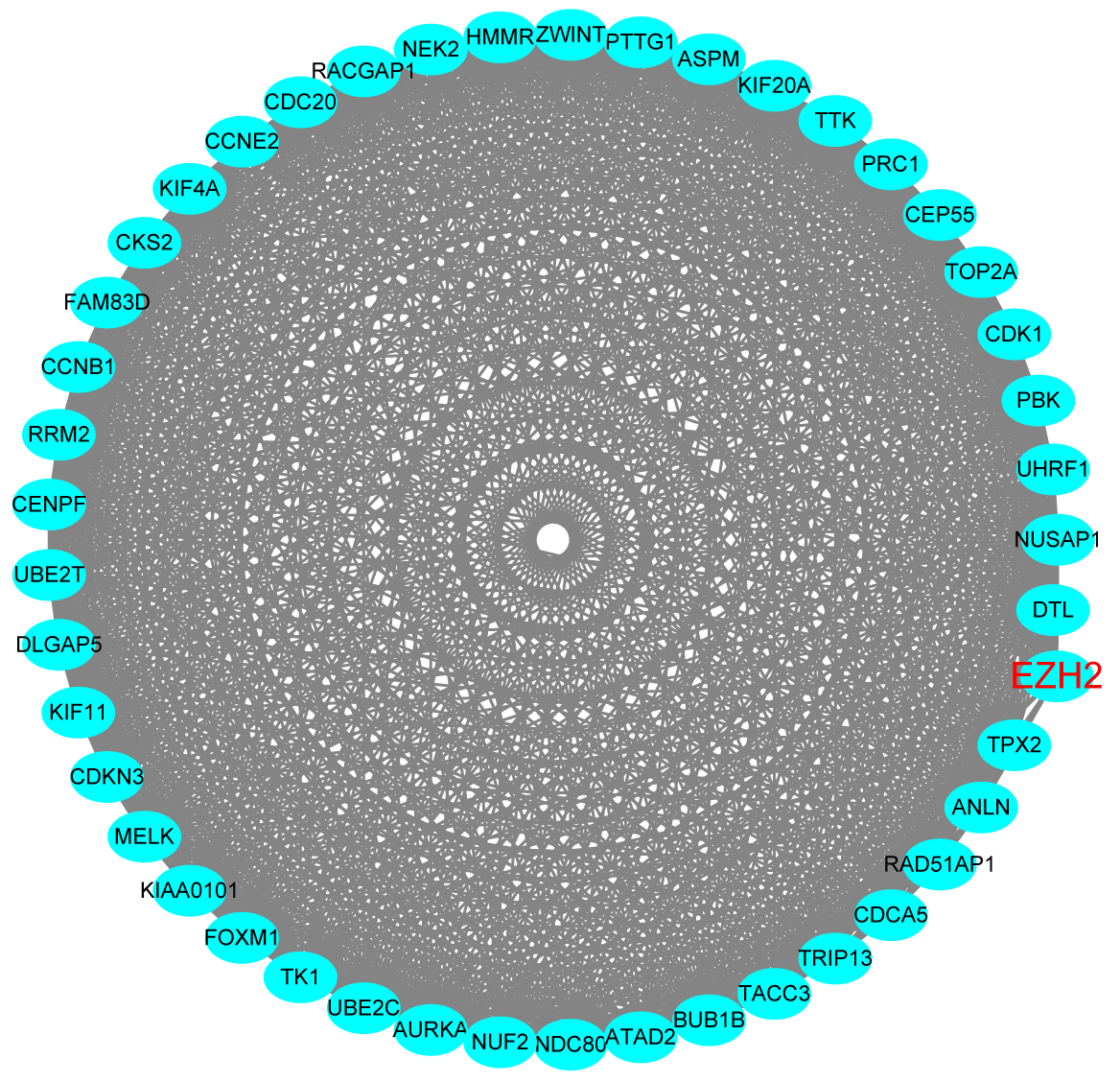

Supplement: Supplementary file 7 — Supplementary Information 7. [file 41598_2021_95068_MOESM7_ESM.tif]

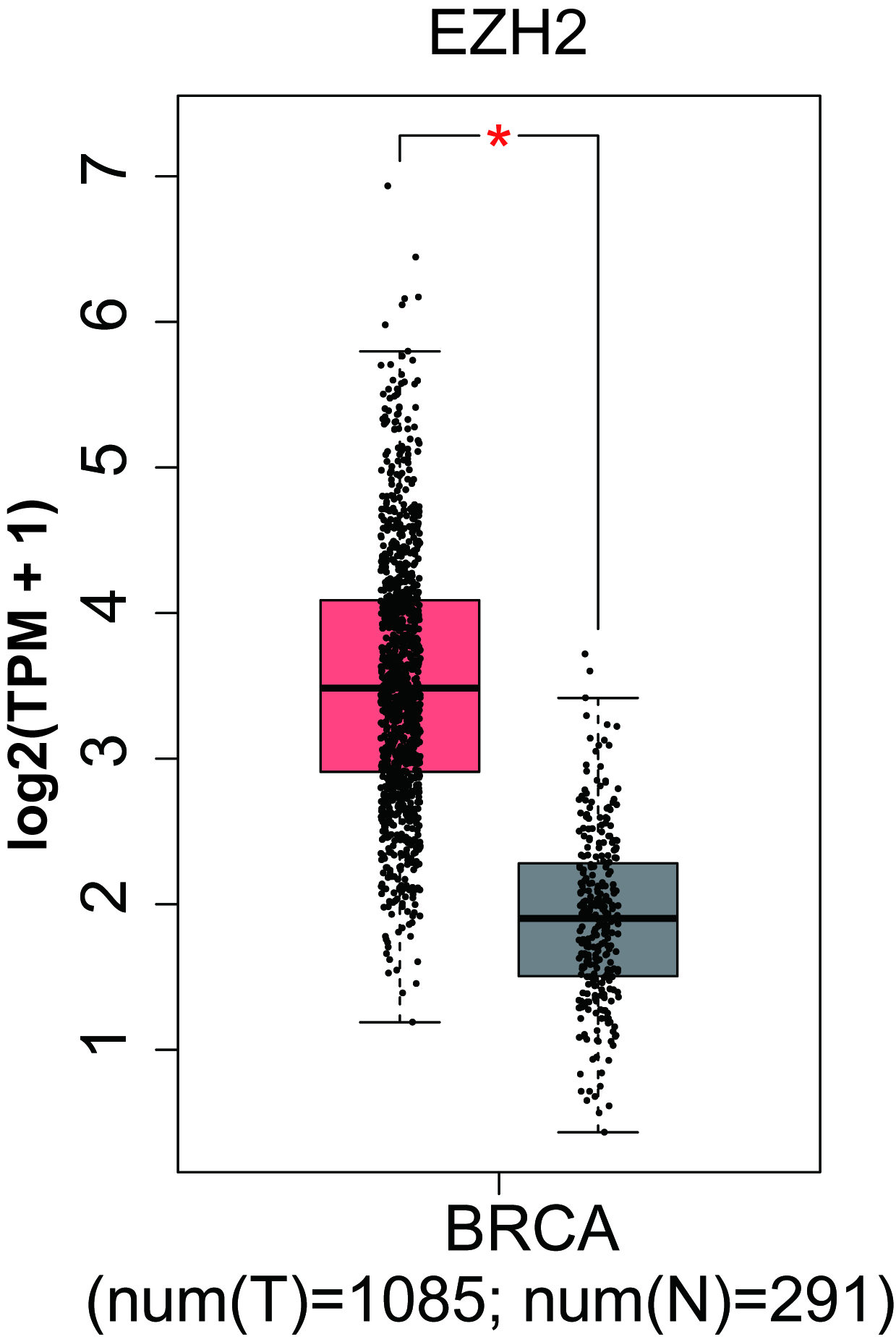

Supplement: Supplementary file 8 — Supplementary Information 8. [file 41598_2021_95068_MOESM8_ESM.tif]

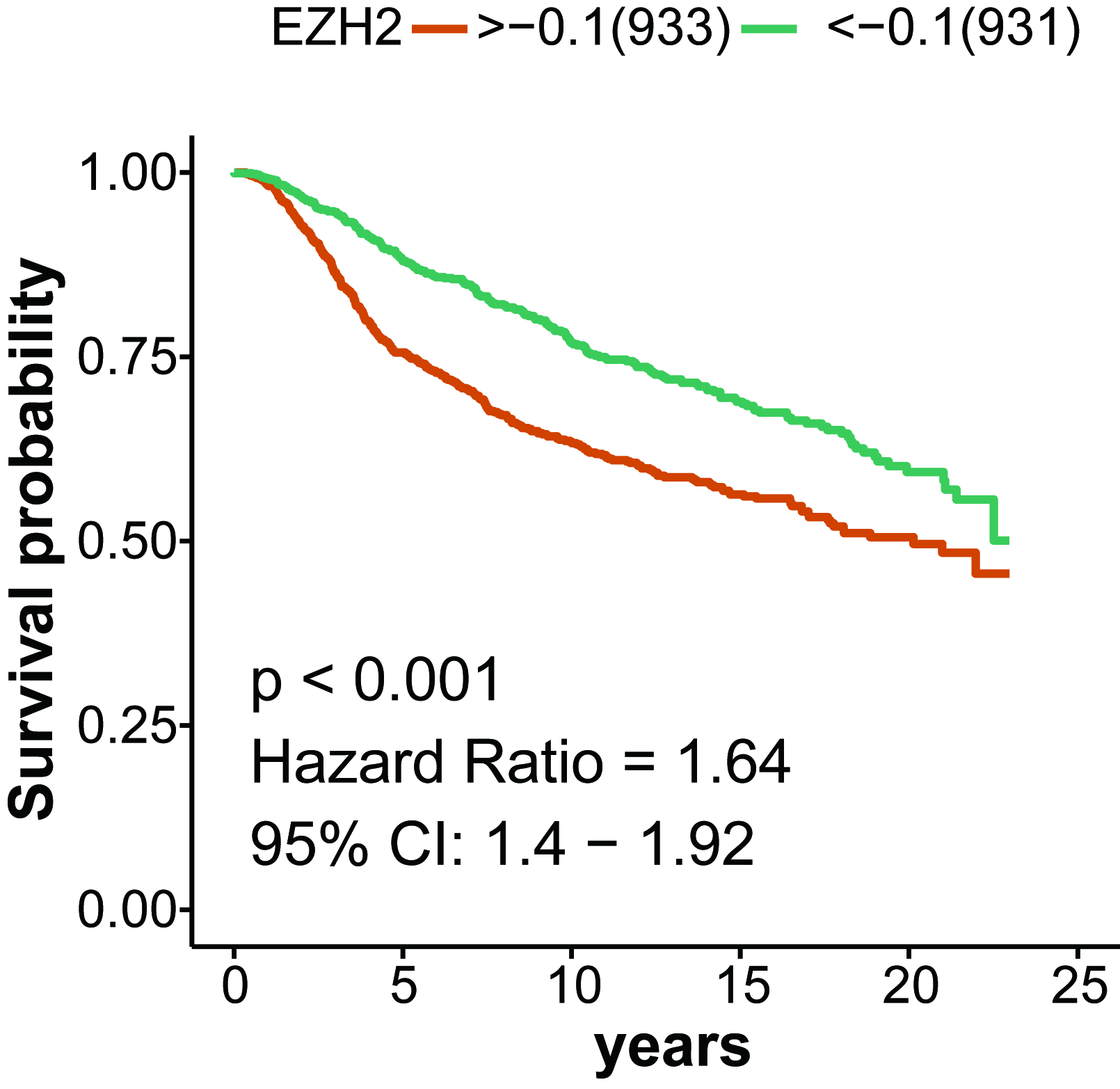

Supplement: Supplementary file 9 — Supplementary Information 9. [file 41598_2021_95068_MOESM9_ESM.tif]

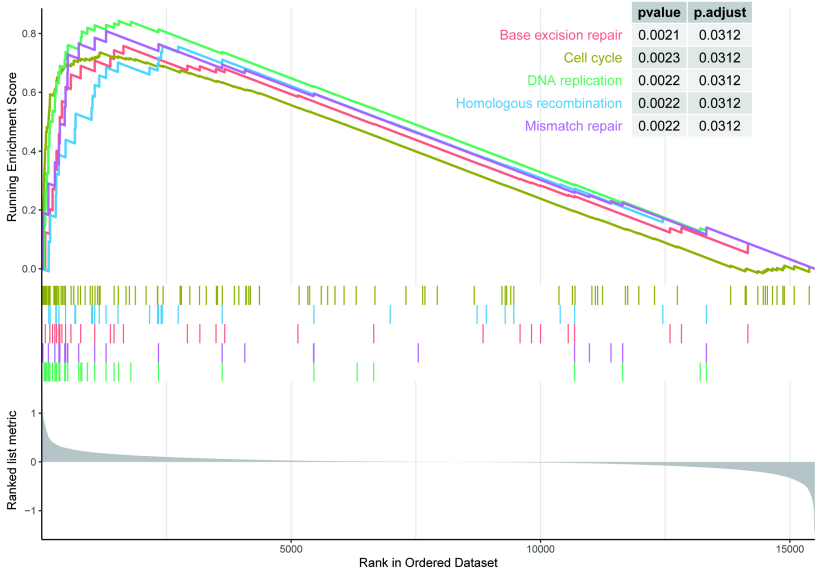

Supplement: Supplementary file 10 — Supplementary Information 10. [file 41598_2021_95068_MOESM10_ESM.tif]

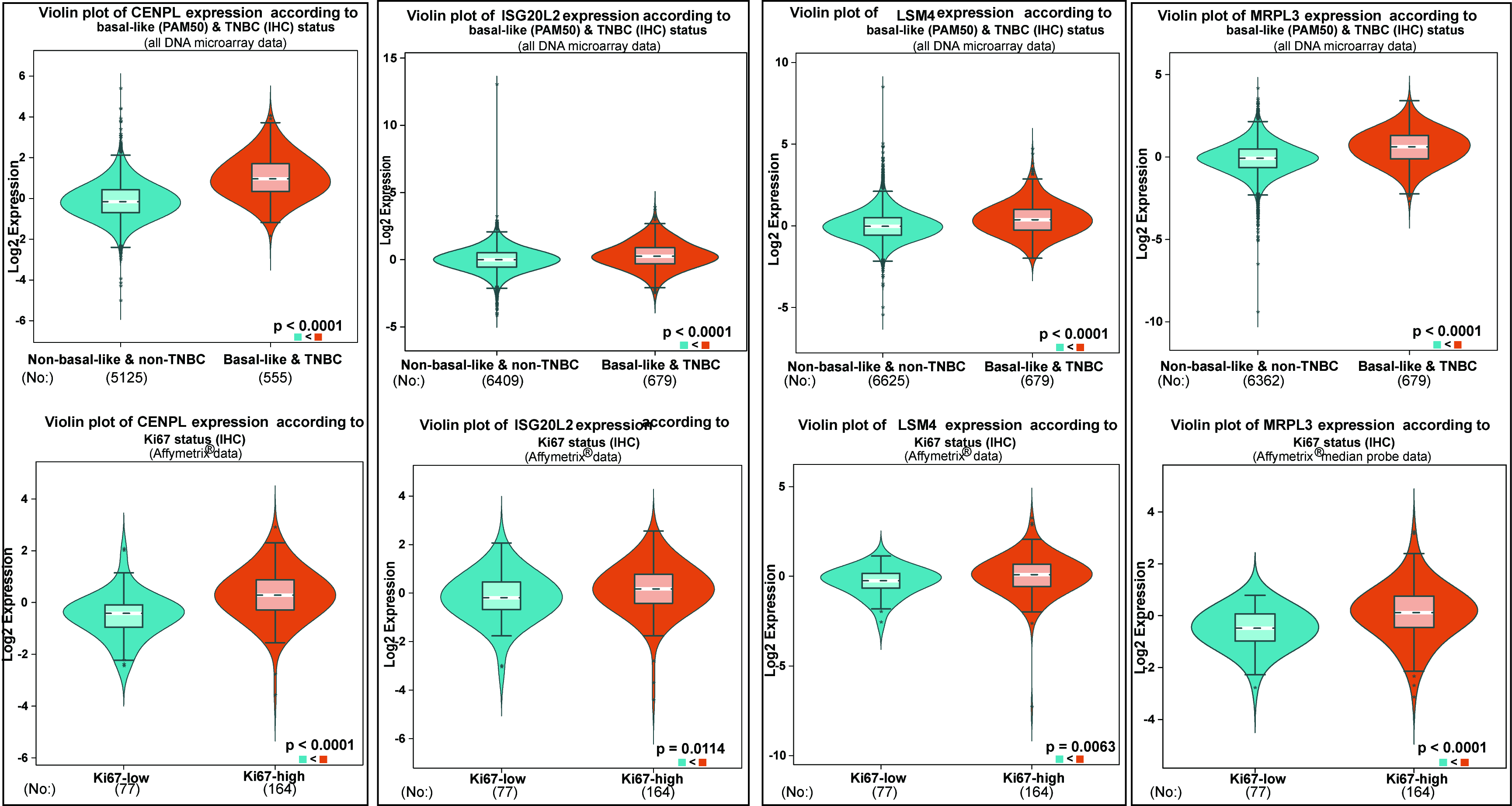

Supplement: Supplementary file 11 — Supplementary Information 11. [file 41598_2021_95068_MOESM11_ESM.tif]

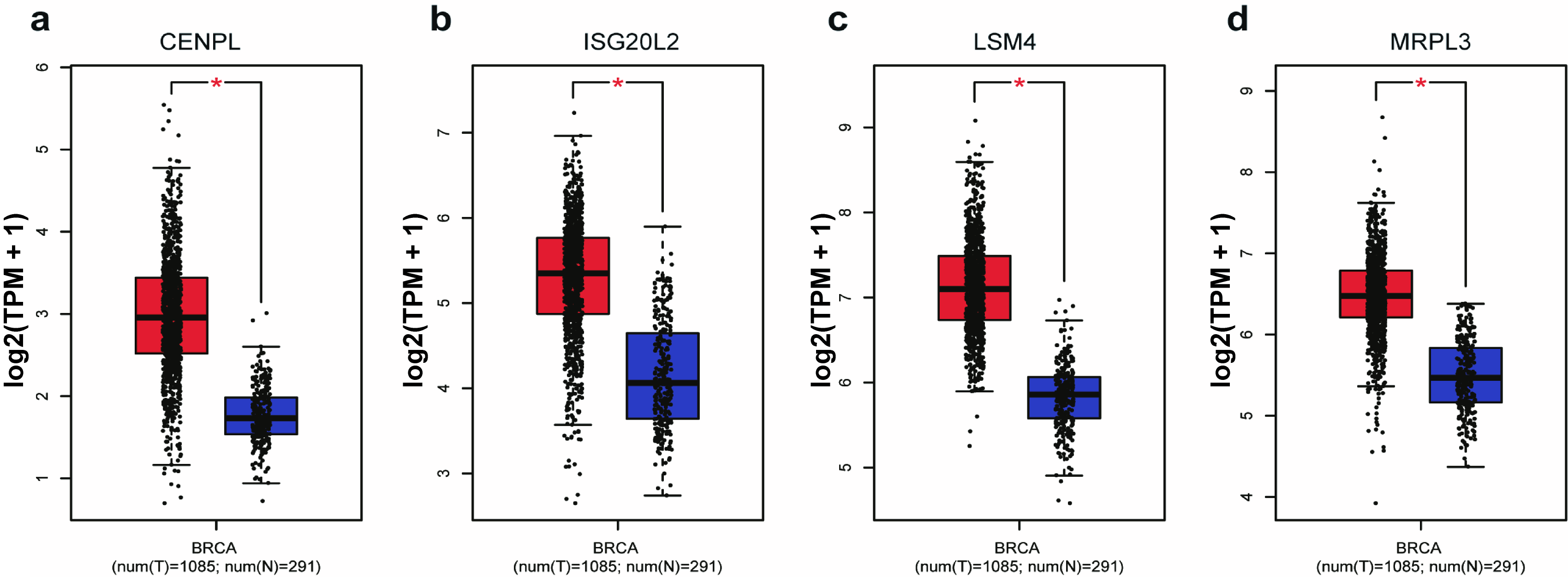

Supplement: Supplementary file 12 — Supplementary Information 12. [file 41598_2021_95068_MOESM12_ESM.tif]

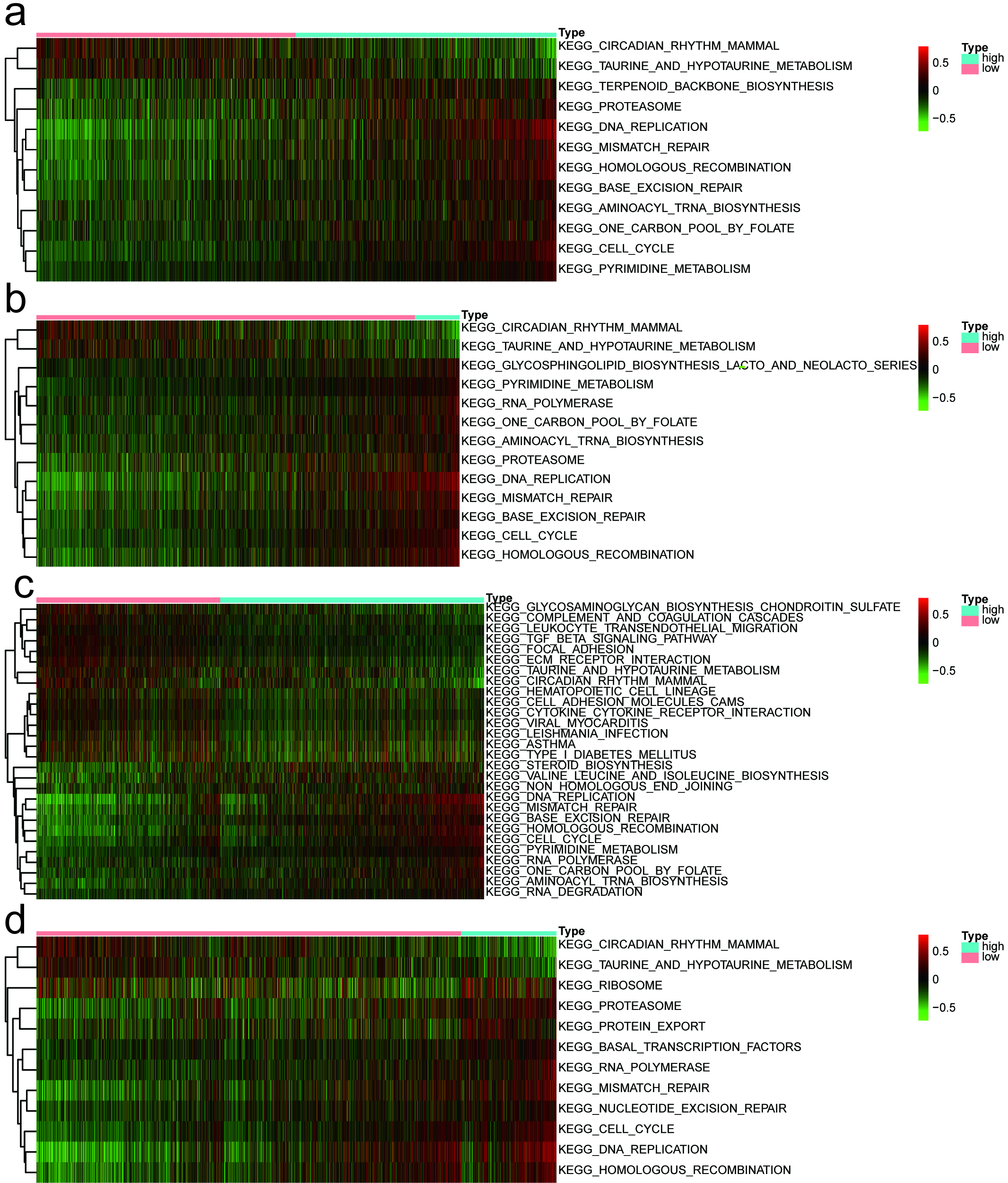

Supplement: Supplementary file 13 — Supplementary Information 13. [file 41598_2021_95068_MOESM13_ESM.tif]

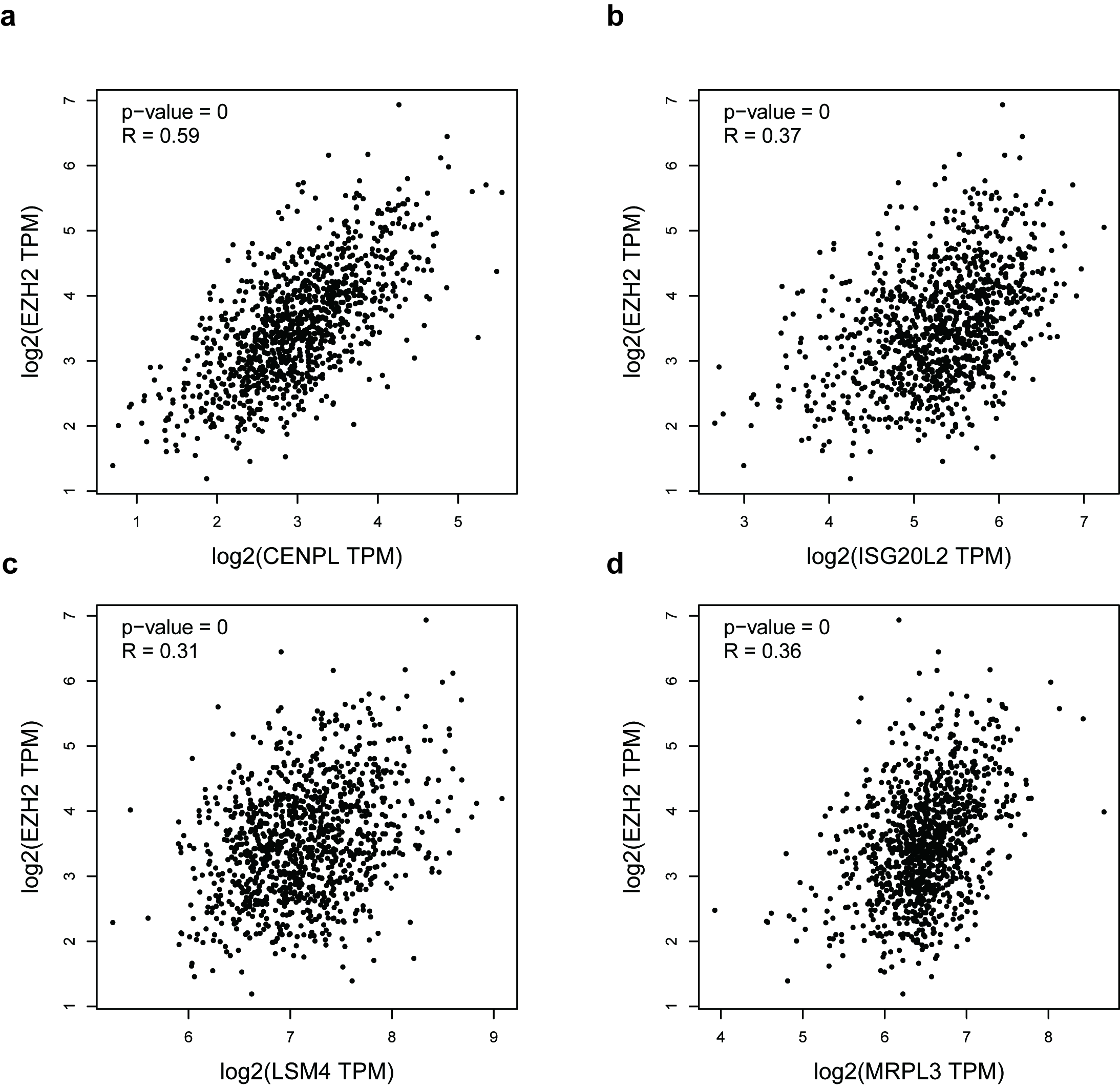

Supplement: Supplementary file 14 — Supplementary Information 14. [file 41598_2021_95068_MOESM14_ESM.tif]
